# Explainable deep learning techniques for microscopic fungi classification using a learnable threshold-based ReLULeaky activation function and transfer learning

**DOI:** 10.1016/j.jpi.2026.100683

**Published:** 2026-06-10

**Authors:** Mohsin Hossain, Mohammad Khairul Islam, Machbah Uddin, Farah Jahan, Md. Shafiul Alam Chowdhury, Md. Ahadullah

**Affiliations:** a Computer Science and Engineering, Uttara University, Dhaka 1230, Bangladesh; b Computer Science and Engineering, University of Chittagong, Chittagong 4331, Bangladesh; c Computer Science and Mathematics, Bangladesh Agriculture University, Mymensingh 2202, Bangladesh

**Keywords:** Fungal infections, DeFungi dataset, ResNet34, Custom ReLULeaky activation function, Deep learning, Transfer learning, ReLU, Leaky ReLU, Fine-tuned model, Learnable threshold, Backpropagation, FastAI library, Explainability, Interpretability, Microscopic Image

## Abstract

Fungal infections are an increasing risk to human health. They can pose a threat to life and cause a variety of health problems. The traditional diagnosis of fungal infections is challenging due to several reasons, such as the lack of clinical mycologists, costly procedures, a high time commitment, and the need for accuracy and specificity requirements. However, early fungal infections detection is essential for effective treatment. In this work, an explainable fine-tuned ResNet34 model for fungi classification is proposed by integrating transfer learning with a learnable threshold-based ReLULeaky activation function to enrich feature representation and classification performance. To improve feature extraction and convergence, the proposed learnable threshold approach dynamically adjusts activation levels during backpropagation. Our proposed fine-tuned ReLULeaky-ResNet34 method outperforms many tests, achieving the best accuracy (95.39%), F1-score (96%), and precision (97%). In addition, the model achieves a 99.40% area under the curve score, ensuring robust classification performance. The study highlights the efficacy of adaptive thresholds by methodically comparing the current and proposed activation functions. Interpretability confirms that the model focuses on biologically significant morphological features. These results demonstrate that our fine-tuned ReLULeaky-ResNet34 model outperforms for accurate and faster fungi classification.

## 1 Introduction

Fungi play a crucial role in recycling and decomposing materials, which helps to keep natural ecosystems in equilibrium.^1^ Since 1969, fungi have been recognized as a distinct group of species with industrial applications, including medicine manufacturing and baking.^2^ It has become widely recognized that some fungi's pathogenic characteristics and propensity to cause disease are crucial to human health. Interestingly, the COVID-19 pandemic has produced important new evidence that emphasizes the effects of bacterial and fungal coinfections in patients who are in life-threatening situations.^3^ Critically ill COVID-19 patients with bacterial or fungal coinfections have significantly longer hospitalizations and a heightened risk of mortality compared to those without such secondary infections.^4^ Another melanized fungus can cause a wide range of illnesses, including systemic infections that can impact the brain, sinuses, and lungs, subcutaneous infections like chromoblastomycosis and phaeohyphomycosis, and superficial infections like tinea nigra.^5^ Invasive fungal infections are a major issue for immunocompromised patients, ranging from colonization to serious systemic infections.^6^, ^7^ Additional risk factors include acute-on-chronic liver failure, corticosteroid use, and gastrointestinal bleeding. The impact is also seen in organ transplant recipients, premature newborns, and critically ill patients.^8^ Prophylactic antibiotic use also contributes to fungal infection susceptibility.^9^ According to recent studies, about 300 million people globally have severe fungal infections yearly, and it is estimated that 1.5 million people lose their lives due to these diseases.^10^ In 1815, mucormycosis, also known as black fungus, was identified. In the past, outbreaks were usually quickly suppressed after being traced to contaminated packaged foods or hospital supplies, but the COVID-19 surge coincided with an abrupt rise in cases in India. Compared to Australia, where the rate is 0.6 per 100,000, the infection rate jumped to 14 per 100,000. Notably, 94% of these cases were linked to individuals with diabetes, highlighting a critical risk factor.^11^ In particular, the rising incidence of invasive fungal infections in immunocompromised people has raised concerns about antifungal resistance.^12^ An expert biologist known as a mycologist makes the diagnosis and classification of a fungal infection in a lab. Patient samples, such as swabs, blood, or skin, hair, or nail swabs, are processed and cultured in controlled media for a time frame of 28–31 days.^13^ Mycologists classify fungi based on their physical characteristics during incubation and growth, allowing dermatologists to treat patients earlier.^14^ Fungal infection diagnosis starts with a direct microscopic examination (DE), magnifying samples up to 1000× without staining. Mycologists identify morphological features to detect yeasts, hyphae, or both. The aforementioned circumstance demands a precise and timely diagnosis of fungal infections. However, conventional diagnostic procedures do have certain limitations as of today. A major drawback of conventional fungal diagnostic techniques is their low sensitivity and specificity, which frequently results in delayed or incorrect identification.^15^ According to conventional methods, the organism is typically cultured on various media, which can be labor-intensive, time-consuming, and frequently produce false-negative results. This leads to severe damage to patients by delaying diagnosis and occasionally yielding incorrect outcomes.^16^ Consequently, a quick and precise way to identify fungal infections is required. This encouraged the authors to investigate fast, high-performing, contemporary, and trustworthy alternatives for the diagnosis of fungal infections. We present a deep learning (DL) framework for the classification of fungal images in this research. Preprocessing and data augmentation are first applied to raw images to improve generalization. A fine-tuned ReLULeaky-ResNet34 model, improved via transfer learning, serves as the architecture's backbone. A learnable threshold is incorporated into a proposedized ReLULeaky activation function within the network, enabling dynamic adaptation based on feature distributions. In more profound ways, this transformation improves non-linearity and learning ability. To ensure transparency in clinical decision-making, explainability techniques are finally deployed at the output to interpret the model's predictions. The results from the proposed method show its outstanding performance in classifying these images.

The key contributions of this research are mentioned below:

• Robust preprocessing and data augmentation to boost model generalization and balance the quantity of the dataset.
• Integration of a learnable threshold during backpropagation into a proposed ReLULeaky activation function to dynamically adapt activation behavior during training, improving non-linearity handling.
• Using transfer learning with a fine-tuned ResNet34 model to improve the classification performance of fungal images.
• Design and evaluate different types of hybrid activation functions to benchmark the effectiveness and performance of our proposed learnable ReLULeaky function.
• Using image explainability methods to ensure model transparency and facilitate clinical decision-making.

The manuscript is structured into five key sections. The first introduces the study, outlining its scope, motivation, and main contributions. The second section reviews related work and existing research in the field. The third section details the proposed methodology and materials employed. The fourth section presents and analyzes the experimental results. Finally, the fifth section concludes the study and discusses potential directions for future research.

## 2 Related works

Convolutional neural networks (CNNs) and DL have been used in the field of microscopic image processing for the classification of fungus species in recent years. In recent studies, the DeFungi dataset^17^ was provided by Sopo et al.,^18^ who also employed VGG16, ResNet50, and InceptionV3 to detect fungal infections initially. In terms of accuracy, VGG16 with transfer learning had the highest score of 85.04%. Tahir et al.^19^ present a new fungal dataset that was developed by collecting images of fungal spores from tainted fruits, archives, and lab-incubated colonies in order to improve fungal detection and classification. 40,800 annotated images of soil and 5 different forms of fungi are included in the entire set. The images, which were labeled using a specific graphical interface, were captured using an optical sensor system. For fungus detection, a CNN-based method was developed, and it achieved 94.8% accuracy. Cuervo et al.^20^ developed a deep neural network-based method in 2019 for determining the different types of Fusarium fungus. Fusarium fungus is a prevalent occurrence in the soil microbiota. Analyzing the infection culture data, their method was able to identify four distinct types of this fungus. In fact, it details a method to identify certain Fusarium species that starts with a microscopic sample image and is based on both artificial neural networks and digital image processing techniques. Although the study focused on images with a high resolution, the model's ultimate accuracy was only 69.51% because of limitations on the amount of test and training data. Hao et al.^21^ employed morphological methods and image preprocessing to build a deep neural network that can identify *Candida albicans* in gynecological tests. Their method achieved 93% accuracy, improving on conventional CNN-based detection. Using the DIFAS dataset, Zielinski et al.^22^ created a CNN-based model that identified 9 different species of Candida fungus with 93% accuracy. Their research improves explainability and usefulness in microbiology by fusing deep neural networks with the bag-of-words method. Using ResNet, Gao et al.^23^ developed an automated microscope to identify fungal infections in samples of skin, nails, and hair. 862 images are used as primary training data for the model. Their model attained recall rates of 99.5% for skin, 95.2% for nails, and 60% for hair, respectively. Mital et al.^24^ evaluated several pretrained CNN models using transfer learning to classify Fusarium fungus species. According to their research, InceptionV2 provides the optimum trade-off between computing efficiency and accuracy. To identify *Cryptococcus neoformans* in microscopic images, Cagatan et al.^25^ designed a DL model based on VGG16. Through web scraping and quality labeling, they generated a dataset that was augmented with data to grow from 126 to 1000 images. The accuracy of the model in classifying images as positive or negative was 86.88%. The potential of DL in fungal diagnosis is shown in this article. Yilmaz et al.^26^ employed grayscale microscopic images to identify onychomycosis, or nail fungus infection, using the VGG16 and InceptionV3 models. A dermatologist classified 297 normal and 160 diseased images in the dataset. Accuracy was 95.98% for VGG16 and 95.90% for InceptionV3. This study shows how well DL works to diagnose fungal infections of the nails. Leveraging meta-evolutionary techniques, Rawat et al.^27^ proposed MeFunX, a DL model for early fungal infection identification in DeFungi microscopic images. Compared to more sophisticated models like VGG16, InceptionV3, and ResNet, it used XGBoost as a learner alongside CNN-based feature extractors. MeFunX showed significant improvements over earlier models with an accuracy of 92.49%. They show how meta-learning may be used to classify fungi. Rahman et al.^28^ employed microscopic images to classify 89 fungal species using a variety of DL models, such as DenseNet, Inception ResNet, and VGG16. To improve training, they applied data augmentation on a dataset of 1079 images. With an average accuracy of 65.35%, DenseNet showed potential in filamentous fungal predictions. This method might shorten the turnaround period and increase diagnostic accuracy. Ahmad and Haque et al.^29^ applied the Vision Transformer (ViT) architecture to look into automated fungal species detection in DeFungi microscopic images. After using transfer learning with pretrained models (VGG16, InceptionV3, and ResNet50), ViT's initial accuracy of 83.12% improved to 90.13%. Their study showed how well ViT captures both local and global visual patterns. Classification performance was significantly improved by fine-tuning using ResNet50. Their method demonstrated improved F1-score, recall, and accuracy. Nawarathne and Kumari^30^ proposed a Big Transfer (BiT) model to classify the fungi spices. Their work addressed class imbalance by data augmentation and preprocessing, using CNN models to classify five different species of fungi. Images were rescaled into 6 different resolutions, and 13 CNN models that had already been trained were used for development. With normalization and a combination of high-resolution and original images, the BiT model achieved the greatest accuracy, 87.32%. Its optimal performance was demonstrated by the least overfitting shown by its loss learning curve. A modified VGG19 DL model for identifying fungal infections from DeFungi microscopic images is presented by Asadi and Seyed et al.^31^ The model outperformed current techniques with an accuracy of 97% by substituting global average pooling (GAP) for the Flatten layer and adding a Dense layer. These results demonstrate how DL may enhance the quality of healthcare and the identification of fungal diseases.

The reviewed study has shown a number of gaps that can be covered using a novel approach. We have proposed a fine-tuned ReLULeaky-ResNet34 model for better feature extraction and classification of fungi classes. Large-scale data augmentation is essential for accurate classification, although it is excluded from many research. A proposed ReLULeaky activation function with a learnable threshold looks promising because traditional activation functions like Rectified Linear Unit (ReLU) and Leaky ReLU established thresholds. It is also possible to optimize typical backpropagation techniques through using learnable threshold values in a proposed activation function. Another gap is the lack of utilization of DL models that are interpretable and explained, which are important for clinical use. Despite the use of transfer learning, feature extraction layer optimization should be improved. Filling up these gaps may result in a DL model for fungal infection diagnosis that is more precise and comprehensible.

## 3 Methods and materials

This section outlines the overall workflow, image preprocessing, and data augmentation techniques used to improve model performance. It also presents a fine-tuned ResNet34 architecture, integrated with a novel ReLULeaky activation function featuring a learnable threshold. Additionally, image explainability methods are applied to ensure transparency and interpretability of predictions.

### 3.1 Workflow of proposed methodology

**Fig. 1:**
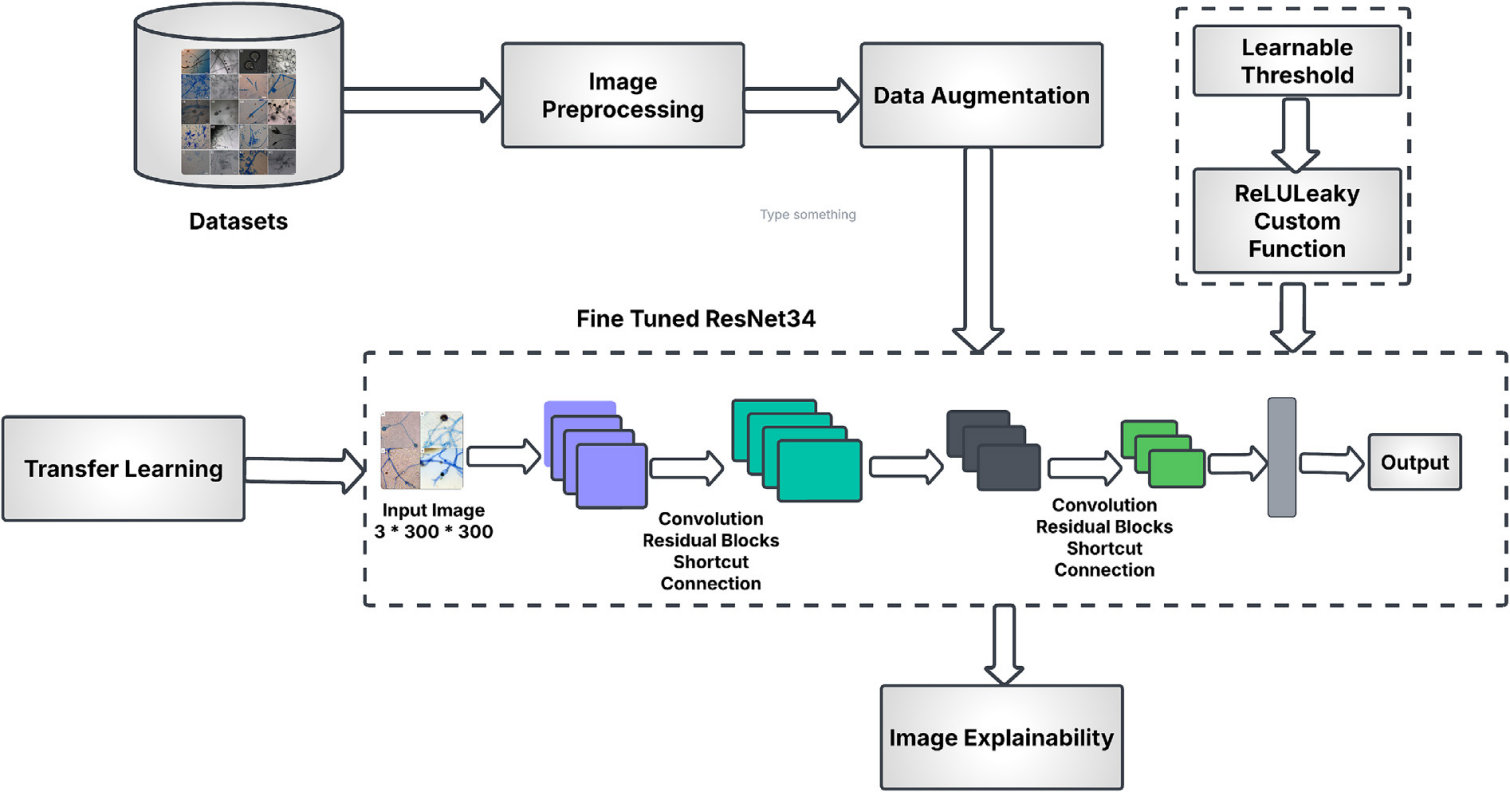
Workflow of the overall study.

This section represents the whole research workflow for fungi classification using a fine-tuned ResNet-34 model with a proposed ReLULeaky activation function and explainability techniques. Fig. 1 depicts the key components of a research workflow, which begins with loading datasets and includes image preprocessing to standardize incoming images through color space transformation, scaling, and normalizing. By using transformations like rotation and flipping, data augmentation improves model generalization and dataset variability. The inclusion of a proposed activation function, ReLULeaky, in place of the conventional ReLU, is a significant change in this model. This function combines ReLU and Leaky ReLU with a learnable threshold. During training, a learnable threshold adjusts to the data to improve classification accuracy by optimizing decision boundaries. The “dying ReLU” issue is mitigated by the ReLULeaky proposed function, a ReLU variant that adds a tiny negative slope for negative inputs. For better results on smaller datasets, the fine-tuned ResNet34 model uses transfer learning and pretrained weights. Convolution residual blocks with shortcut connections handle vanishing gradients by adding input directly to output, allowing deeper networks, whereas input images are scaled to 300 × 300 pixels for uniformity. Convolutional feature extraction is followed by a fully connected layer that transforms the processed features into a classification output. This result, which identifies the most likely class for a given input, shows the probability distribution between fungi classes. Finally, the explainability and interpretability of images are incorporated into the model, which improves decision-making transparency.

### 3.2 Dataset descriptions

The Laboratory of Electron Microscopy and Microanalysis (LEMM) of Columbia graciously provided the dataset for this study, which comprises a set of microscopic fungal images. The entire set initially comprised 3000 images without any curation or annotations. Several thoroughly executed processes were performed to prepare the dataset for analysis.^18^ The images were initially resized into 3×500×500 from their original dimensions to ensure uniformity across the dataset. Each image was then patched after being resized, creating a total of 88 patches for every single image. For the purpose of improving the dataset's quality and relevance, a thorough manual inspection was done to find and remove patches that were thought to be unreliable or contained distortion. This rigorous selection procedure made sure that only patches relevant to the classification task were preserved. The 50 patches were chosen from each image following the manual evaluation as a final refinement. The goal of this selection was to keep the changes that were the most informative while getting rid of redundant code. After the wrapping up of these preparation operations, a final dataset including 9114 images was produced. This assortment shows how well-integrated, scaled, curated, and hand-picked patches from the original, uncurated images.

This dataset contains five different types of fungi, as follows: *Candida. albicans* (H1), *Aspergillus niger* (H2), *Trichophyton rubrum* (H3), *Trichophyton mentagrophytes* (H5), and *Epidermophyton floccosum* (H6).

**Fig. 2:**
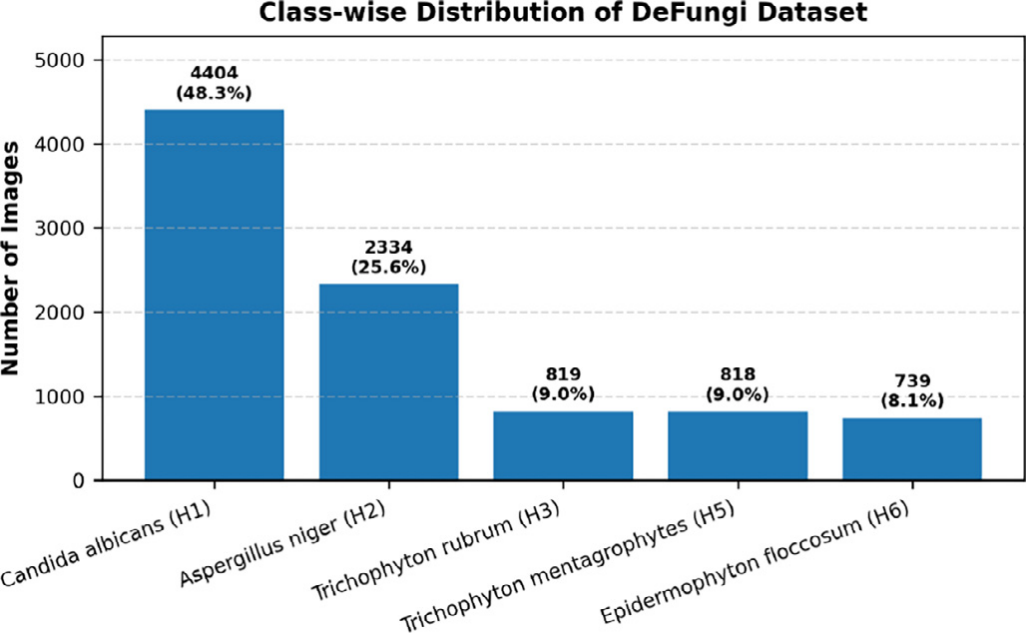
Class-wise distribution of the DeFungi dataset.

Fig. 2 depicts the class distribution of the DeFungi dataset. As observed, the images are evenly distributed among the classes, indicating a well-balanced dataset. This balanced distribution reduces the likelihood of model bias toward dominant classes and contributes to more robust training, enhanced generalization, and equitable performance evaluation across all fungal species.

**Fig. 3:**
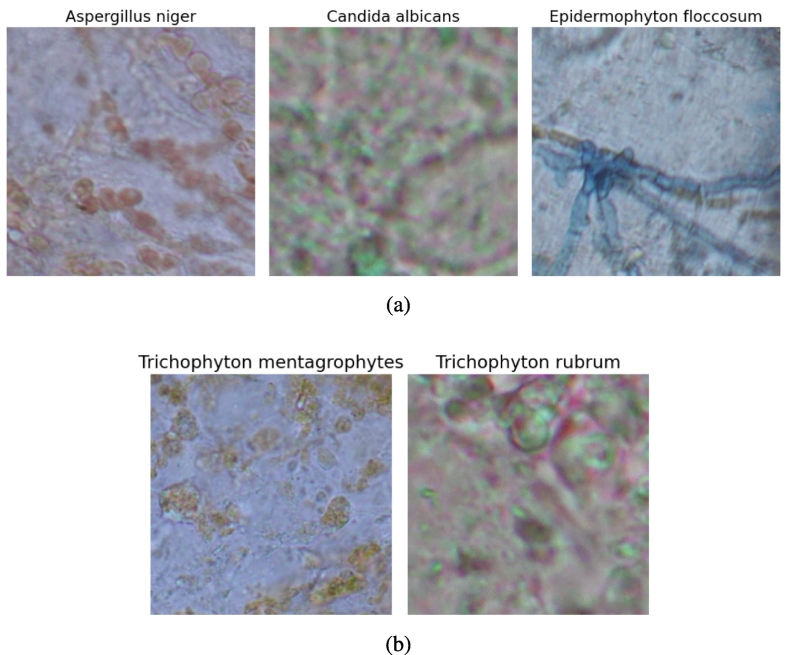
Representative microscopic images of the five fungal classes used in this study. The classes are distinguished by their characteristic microscopic morphology, including variations in hyphal structure, spore arrangement, branching patterns, and cellular texture, which provide discriminative features for DL-based classification.

Fig. 3 shows exemplary microscopic images of the five fungal species. Fig. 3a presents *A. niger* (H2), *C. albicans* (H1), and *E. floccosum* (H3), highlighting their unique morphological features, such as dark conidial heads, oval yeast cells with pseudohyphae, and club-shaped macroconidia, respectively. Fig. 3b depicts *T. mentagrophytes* (H5) and *T. rubrum* (H6), which are distinguished by spiral hyphae with clustered microconidia and elongated septate hyphae with tear-shaped microconidia, respectively.

### 3.3 Image preprocessing

In a DL image classification pipeline, several crucial preprocessing steps are employed before feeding the images into the model. The first step is resizing, which ensures all input images have a consistent size, facilitating batch processing and efficient GPU utilization. The following mathematical relationships are used throughout the resizing process:

(1) $$X=\frac{x}{W_{\text{original}}}\times W_{\text{resized}}$$

(2) $$Y=\frac{y}{H_{\text{original}}}\times H_{\text{resized}}$$

The dimensions of the original and scaled versions are $W_{\text{original}}\times H_{\text{original}}$ and $W_{\text{resized}}\times H_{\text{resized}}$. Both of them are $\left(x,y\right)$ and $\left(X,Y\right)$, the original and scaled pixel values. From the original 3×500×500 form, we reduced the images to 3×300×300. For every value of pixels, the scaling process is interpolated. It uses fungal image databases to ensure a standard input size and reduce computational complexity.

Following resizing, the images are converted into tensors for computation with PyTorch. The tensor is denoted $T\in R^{c\times H\times W}$, reordering dimensions to match PyTorch's channel-first format. This tensor also typically feeds the normalization:

$$T^{\prime }=\frac{T-\mu }{\sigma }$$

**Fig. 4:**
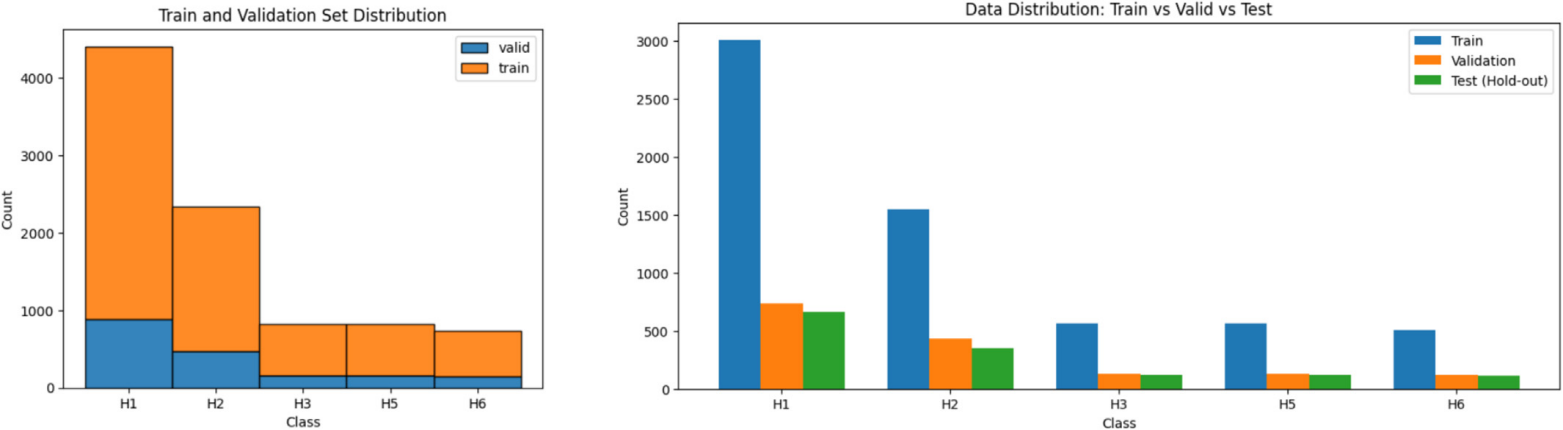
Data splitting distribution.

Where μ and σ are the mean and standard deviation of pixel intensities, usually precomputed from the dataset itself. The next step, label extraction, involves determining the class for each image. This is commonly done by extracting the name of the parent directory that contains the image files. In this study, two different data-splitting strategies (as illustrated in Fig. 4) were employed to comprehensively evaluate the performance of the proposed fungi classification models. Using the FastAI framework, the dataset was divided into 80% training and 20% validation, as supported by the *ParentSplitter*, to analyze training performance. This split was primarily used to analyze model learning behavior and convergence during training. Additionally, a three-way split was implemented using TensorFlow, where 76.5% of the data were used for training, 13.5% for validation, and 10% for testing. This additional setup enabled an independent evaluation of the model's generalization performance.

### 3.4 Data augmentation

Data augmentation is a key approach in machine learning and DL that artificially enriches and diversifies training datasets to improve model performance. It involves using transformations like rotation, scaling, flipping, and noise addition to generate new data while keeping essential features. Augmentations involve geometric and color adjustments. The goal is to improve generalization and prevent overfitting, specially for small or imbalanced datasets. By exposing the model to varied inputs, it learns more robust features. Data augmentation is typically employed in real time during training, making it an efficient, cost-effective way to boost model accuracy without requiring more labeled data.

Vertical flipping randomly flips the image along the vertical axis with a probability of 0.5. This can be represented as:

$$I^{\prime }=\left\{\begin{array}{cc} \text{flip}\left(I,\text{axis}=y\right) & \text{with probability}\;0.5\\ I & \text{otherwise}. \end{array}\right)$$

Images are randomly rotated up to a maximum of ±15${}^{{}^{\circ}}$. This rotation is applied using a uniform random distribution within the specified range:

$$I^{\prime }=\text{rotate}\left(I,\theta \right)\;\text{where}\;\theta ∼U\left(-15^{{}^{\circ}},15^{{}^{\circ}}\right).$$

The zoom transformation scales the image by a factor randomly chosen between 1 and 1.3. This is equivalent to:

$$I^{\prime }=\text{zoom}\left(I,z\right)\;\text{where}\;z∼U\left(1.0,1.3\right).$$

Lighting transformations adjust the brightness and contrast of the image. With a probability of 0.75, the brightness and contrast of the image are adjusted by a factor up to 20%.

**Fig. 5:**
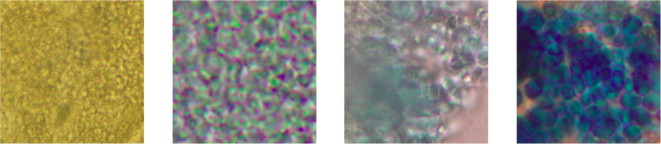
Original images.

**Fig. 6:**
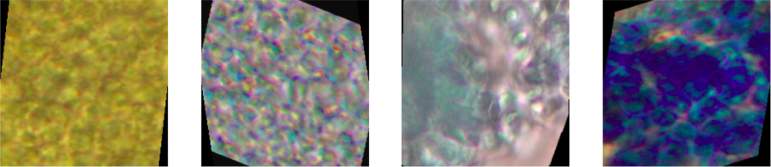
After augmentation.

Fig. 5 depicts the fungal original images, whereas Fig. 6 illustrates the images after they have been augmented. A number of variations are made to the images, such as zooming, random rotation, vertical flipping, and lighting alterations. To improve the diversity of the training dataset and strengthen the robustness of the model, these augmentations are applied probabilistically.

### 3.5 Proposed fine-tuned ResNet34 model

In our proposed method, we fine-tune a ResNet-34 CNN, a deep residual network known for its potent performance in image classification tasks. This model is pretrained on a large dataset (like ImageNet), enabling it to leverage rich, general-purpose image features. We adapt it to our specific fungi classification task through fine-tuning, which involves updating the pretrained weights using our own training data.

**Fig. 7:**
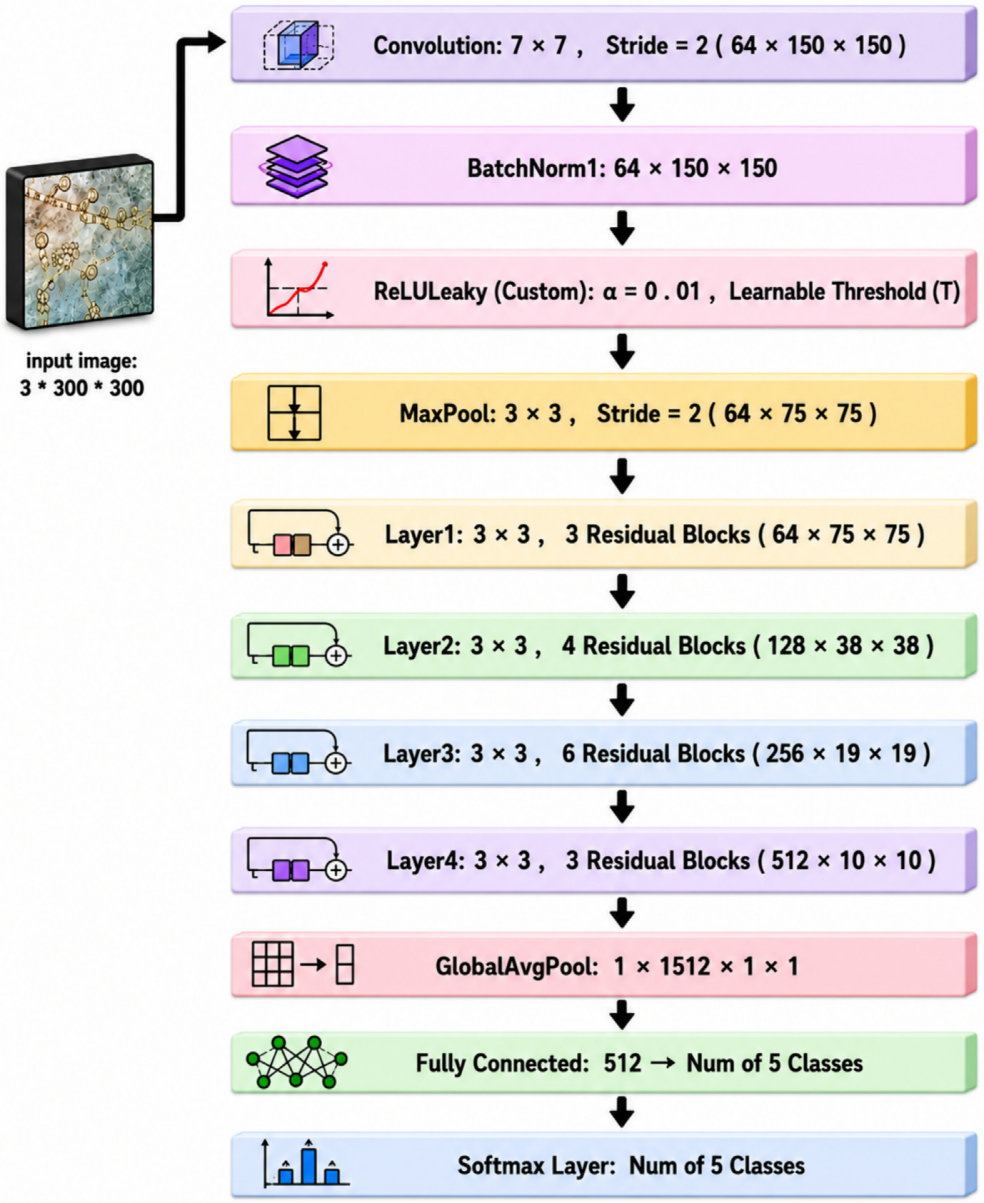
Fine-tuned ResNet34 architecture with ReLULeaky activation function.

Fig. 7 represents the fine-tuned ResNet-34 architecture, incorporating the proposed activation function ReLULeaky with a learnable threshold and a fixed slope parameter (α=0.01). The model begins with an Input Layer processing 3×300×300 images, followed by a convolutional layer with a 7×7 kernel and stride 22, and a Batch Normalization step. The proposed ReLULeaky function replaces standard ReLU in all residual blocks, enabling learnable parameters to adapt activation behavior dynamically. A MaxPooling Layer reduces the spatial dimensions before feeding into the residual Layer1 through Layer4, which progressively increase feature maps (64−>512) while reducing tensor dimensions. Each layer consists of stacked residual blocks with hyperparameters tuned for depth (3,4,6) and kernel size 3×3. Tuning hyperparameters is crucial for performance optimization. For training, the one-cycle policy is being used. Throughout the training process, this method continually adjusts the learning rate to boost generalization and faster convergence. It starts at a lower learning rate, climbs slowly to a peak value in the first half of the training, and then declines once more in the later portion. By using a cyclic policy, the model stays robust and avoids getting stuck in local minima. In order to prevent quick weight updates and promote smoother optimization, the one-cycle policy also includes momentum scheduling, in which momentum is inversely correlated with learning rate, with higher momentum during lower learning rates and vice versa. Initially, the model is trained using a one-cycle policy for five epochs, and then it is fine-tuned. While preventing overfitting, these methods enable the gradual unfreezing of deeper layers to accommodate fungi classification. By leveraging transfer learning, hyperparameter tuning, and proposed ReLULeaky activation functions, the complete fine-tuning process ensures that the ResNet34 model is ideally suited for fungi classification.

### 3.6 Proposed ReLULeaky activation function

The ReLULeaky (a proposed activation function) is designed to combine the benefits of the ReLU^32^ and leaky ReLU^33^ with a learnable threshold parameter. By adding a learnable threshold for the activation, the aim is to allow small negative values during the forward pass. This allows the network to better handle cases, where the standard ReLU (which zeros out negative values) might cause issues in deeper networks, especially during training. The proposed function combines the ReLU operation with a learnable threshold and Leaky ReLU behavior for values below this threshold. The function is adaptive, meaning the threshold is adjusted during training to better fit the data. The ReLU function give outputs zero for negative values and passes positive values unchanged. However, this can cause the dying ReLU problem, where some neurons become inactive and stop contributing to learning. Leaky ReLU addresses this issue by allowing small negative values instead of zero, using a slope for negative inputs. The ReLULeaky function further extends this idea by introducing a learnable threshold *T*, ensuring flexibility in defining the activation boundary. The ReLULeaky activation function can be defined as:

$$f\left(x\right)=\left\{\begin{array}{cc} x & \text{if}\;x>T\\ \mathrm{\alpha x} & \text{if}\;x\leq T \end{array}\right)$$

Where x is the tensor (features from the neural network). Threshold (*T*) is a learnable parameter initialized with a value, typically 0.1, and α (often small, like 0.01) is the slope of the negative part, a standard parameter for the Leaky ReLU component. The ReLULeaky activation functions algorithm 1 is written below:

Algorithm 1 Proposed ReLULeaky activation function.

**Figure:**
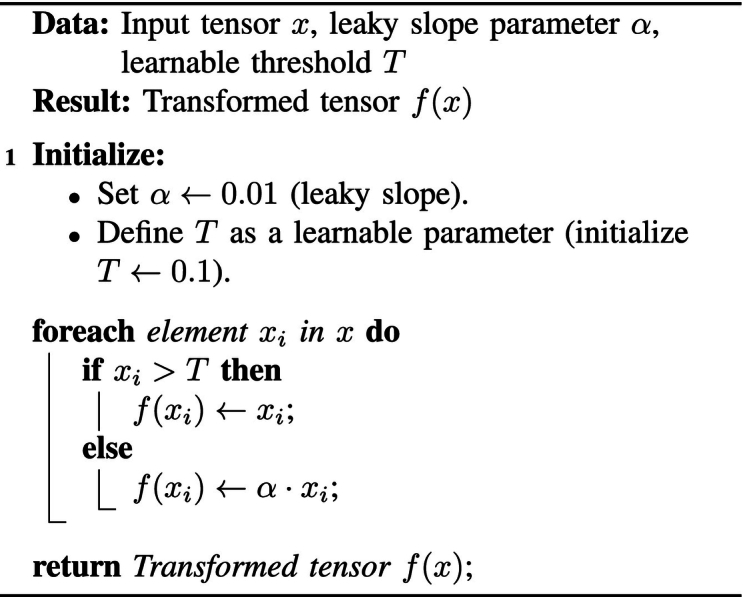

This algorithm 1 defines a proposed ReLULeaky activation function with a learnable threshold T. During the forward pass, each input element is passed unchanged if it exceeds T otherwise, it is scaled by a leaky factor α. In the proposed framework, the threshold value T is modeled as a trainable parameter and learned via backpropagation at each layer. Gradient updates for T are derived during the backward pass and optimized concurrently with the network parameters. This approach allows the activation function to dynamically adapt its threshold during training. The detailed backpropagation algorithm 2 used to compute the threshold value T is presented below.

Algorithm 2 Backpropagation of learnable threshold (*T*).

**Figure:**
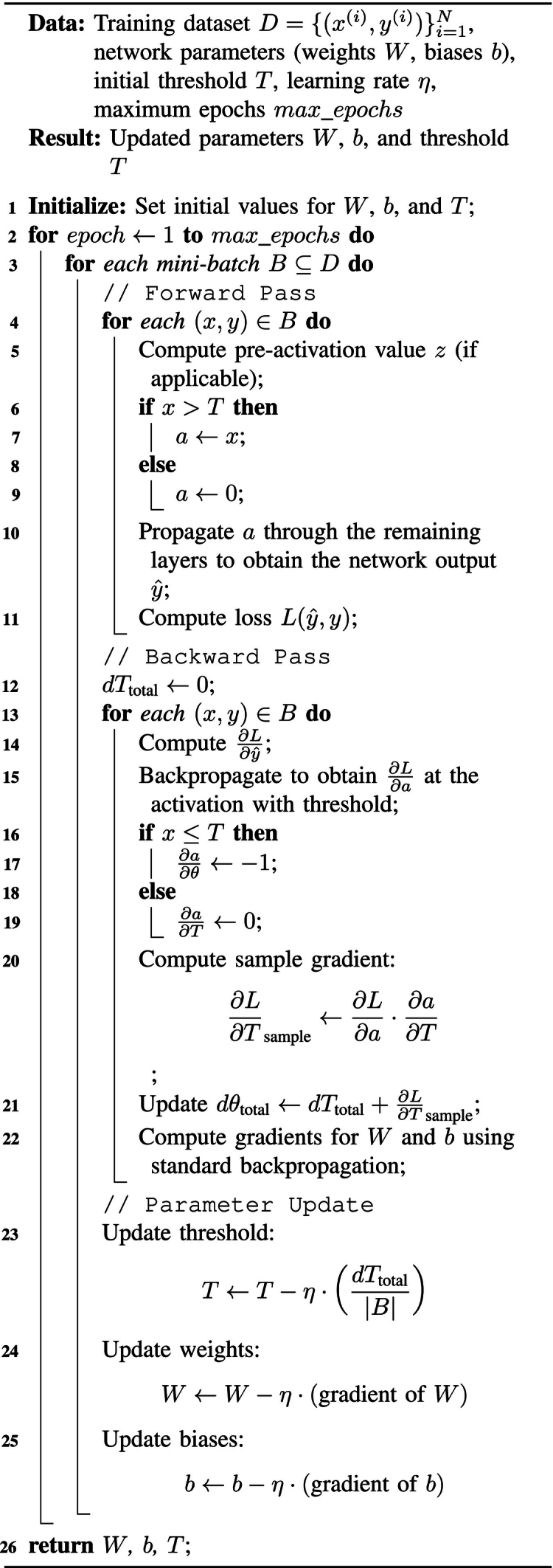

• **Initialization (Lines 1–2):** The network weights W, biases b, and the learnable threshold T are initialized. Making T learnable allows the network to adaptively determine the point at which the activation “turns on.”
• **Forward pass (Lines 4–9):** For each training sample, the activation function is applied:
  $$a=\left\{\begin{array}{cc} x, & \text{if}\;x>T,\\ 0, & \text{if}\;x\leq T. \end{array}\right)$$

The output is then propagated to compute the network prediction $\hat{y}$, and a loss function $L\left(\hat{y},y\right)$ (e.g., mean-squared error prediction) is evaluated.

• **Backward pass (Lines 10–15):** Gradients are computed with respect to the network parameters and the threshold T. The derivative of the activation with respect to T is:
  $$\frac{\partial a}{\partial T}=\left\{\begin{array}{cc} -1, & \text{if}\;x\leq T,\\ 0, & \text{if}\;x>T. \end{array}\right)$$

This is used (via the chain rule) to compute the gradient $\frac{\partial L}{\partial \theta }$ for each sample, which is then accumulated over the mini-batch.

• **Parameter update (Lines 16–17):** The threshold, weights, and biases are updated using gradient descent. For T, the update is:
  $$T\leftarrow T-\eta \cdot \left(\frac{1}{|B|}\sum_{\left(x,y\right)\in B}\frac{\partial L}{\partial a}\cdot \frac{\partial a}{\partial T}\right).$$

Algorithm 3 Replacement of ReLU layers with proposed ReLULeaky activation function.

**Figure:**
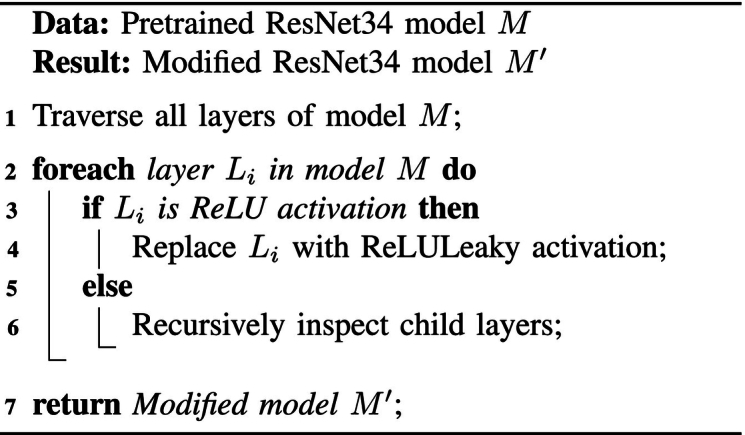

Algorithm 3 demonstrates the recursive replacement method of conventional ReLU activation layers with the proposed learnable threshold-based ReLULeaky activation function within the pretrained CNN architecture. Initially, the pretrained ResNet34 model M is traversed layer-by-layer to recognize all typical ReLU activation layers. Although ReLU is computationally efficient, it may suffer from the dead neuron problem due to complete suppression of negative activations. To remedy this limitation, each recognized ReLU layer $L_{i}$ is replaced with the proposed ReLULeaky activation function. Unlike classic ReLU, the proposed activation retains weak feature responses through non-zero activation in low-response regions while adaptively learning the activation boundary during optimization. A ResNet34 model contains nested residual blocks and hierarchical convolutional modules, the algorithm recursively examines all child layers to assure complete activation replacement across the network. After replacement, the modified model can be stated as:

$$M^{\prime }=\left\{L_{1}^{\prime },L_{2}^{\prime },L_{3}^{\prime },\ldots ,L_{n}^{\prime }\right\}$$

where:

$$L_{i}^{\prime }=\left\{\begin{array}{ll} \text{ReLULeaky}, & \text{if}\;L_{i}=\text{ReLU}\\ L_{i}, & \text{otherwise}. \end{array}\right)$$

This replacement approach enhances feature propagation, stabilizes gradient flow, and retains discriminative microscopic texture information during deep feature extraction.

Algorithm 4 Training procedure using ReLULeaky activation function.

**Figure:**
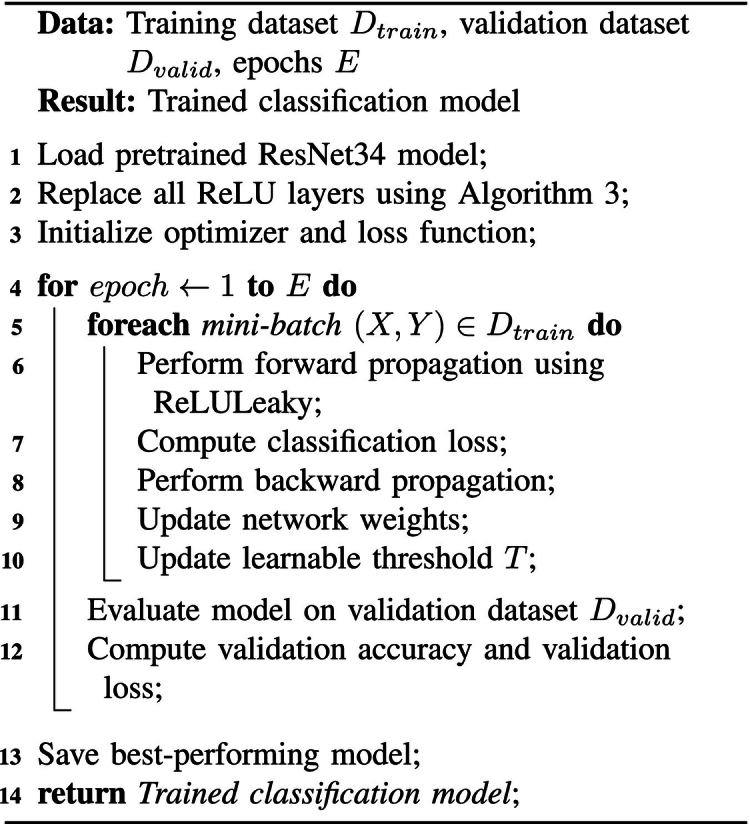

Algorithm 4 presents the training workflow of the proposed ReLULeaky-based ResNet34 model for microscopic fungi image classification. The pretrained network is first updated by replacing all ReLU layers with the proposed learnable threshold-based ReLULeaky activation function. During training, forward propagation, loss computation, and backpropagation are performed to update both network weights and the learnable threshold parameter T. The model is then assessed on the validation dataset after each epoch.

Algorithm 5 Batch-wise activation computation.

**Figure:**
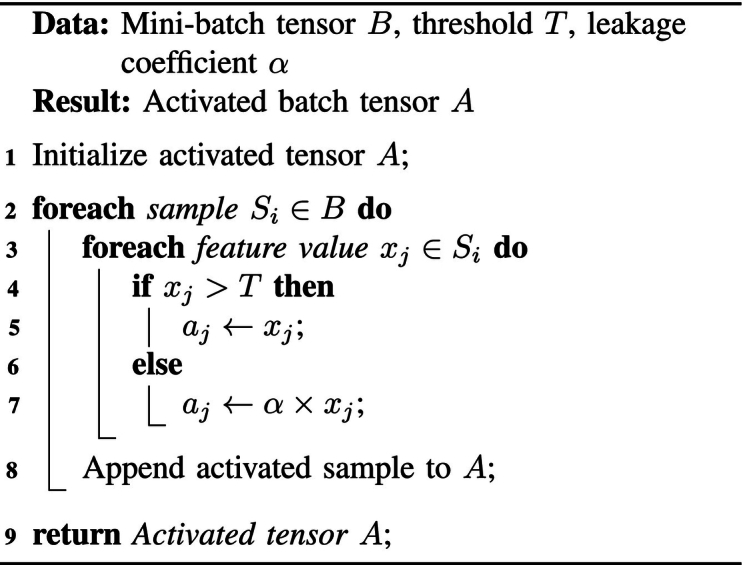

Algorithm 5 outlines the batch-wise activation computation using the proposed ReLULeaky activation function. For each feature value $x_{j}$ in the mini-batch tensor B, the activation is estimated as follows:

$$f\left(x\right)=\left\{\begin{array}{cc} x, & x>T\\ \mathrm{\alpha x}, & x\leq T. \end{array}\right)$$

This batch-wise activation process retains weak feature responses, increases gradient propagation, and boosts discriminative feature learning during deep network training.

Algorithm 6 Gradient flow preservation in ReLULeaky.

**Figure:**
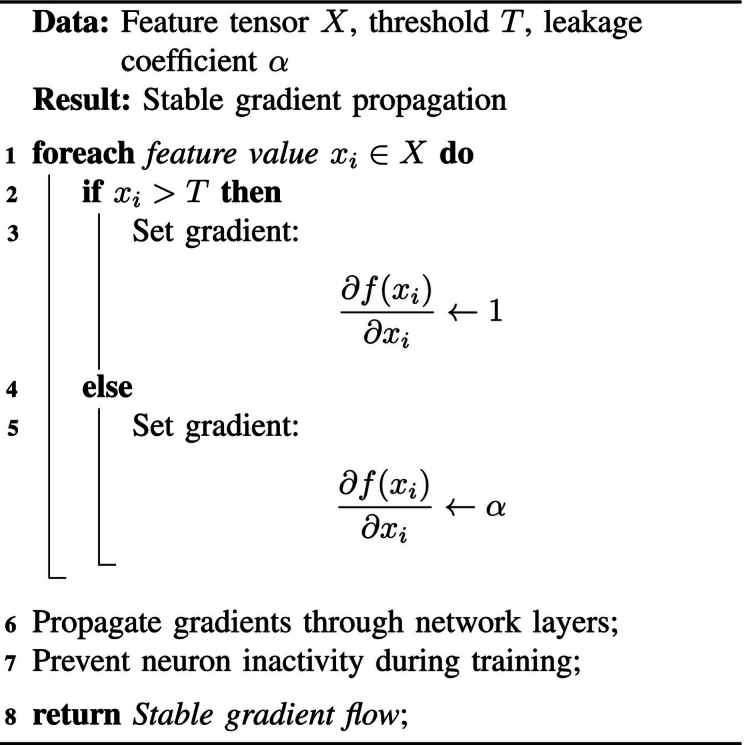

Algorithm 6 explains the gradient propagation mechanism of the proposed ReLULeaky activation function. Unlike standard ReLU, the proposed activation keeps non-zero gradients in low-response regions, which enhances gradient flow, reduces neuron inactivity, and stabilizes deep network training.

Algorithm 7 Feature propagation using Learnable Threshold ReLULeaky activation function.

**Figure:**
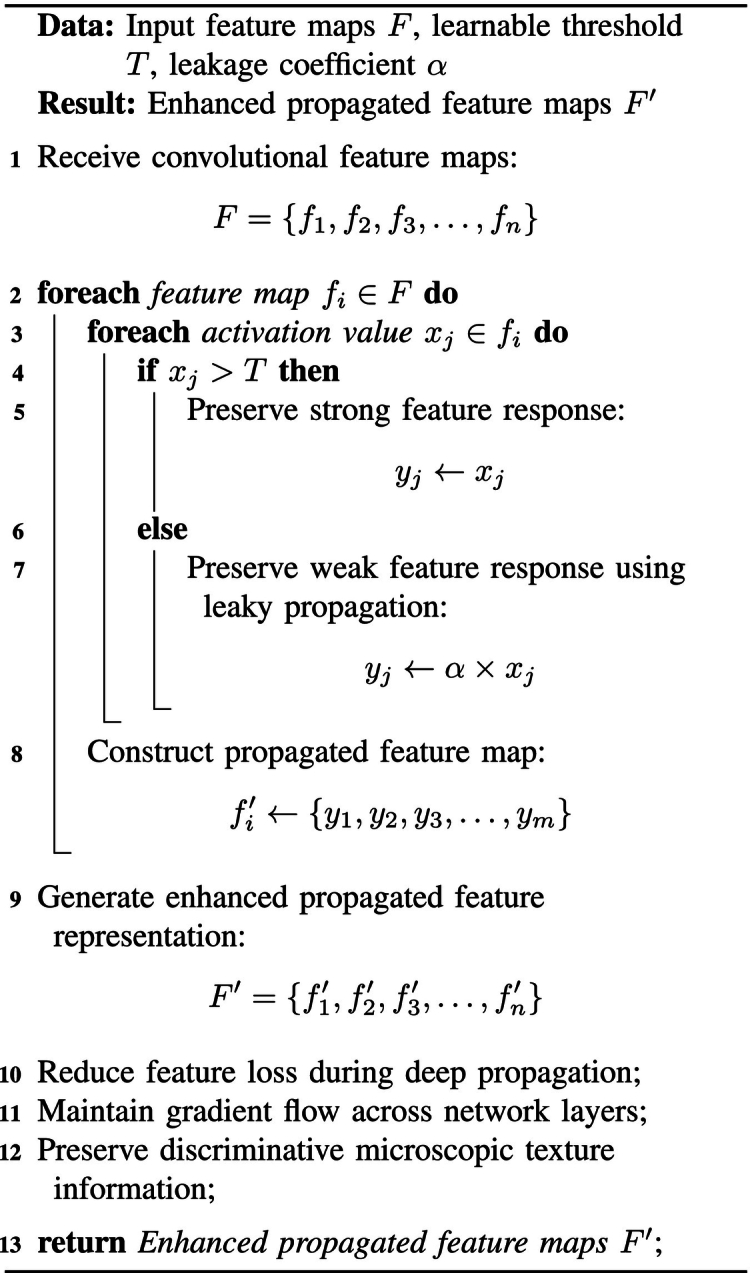

Algorithm 7 shows how the proposed ReLULeaky activation propagates feature information across the network. Each feature value is evaluated against the learnable threshold T, with strong activations kept and weaker activations retained through a leakage coefficient α. This process reduces feature loss, maintains informative microscopic texture patterns, and facilitates effective feature propagation for accurate classification.

**Fig. 8:**
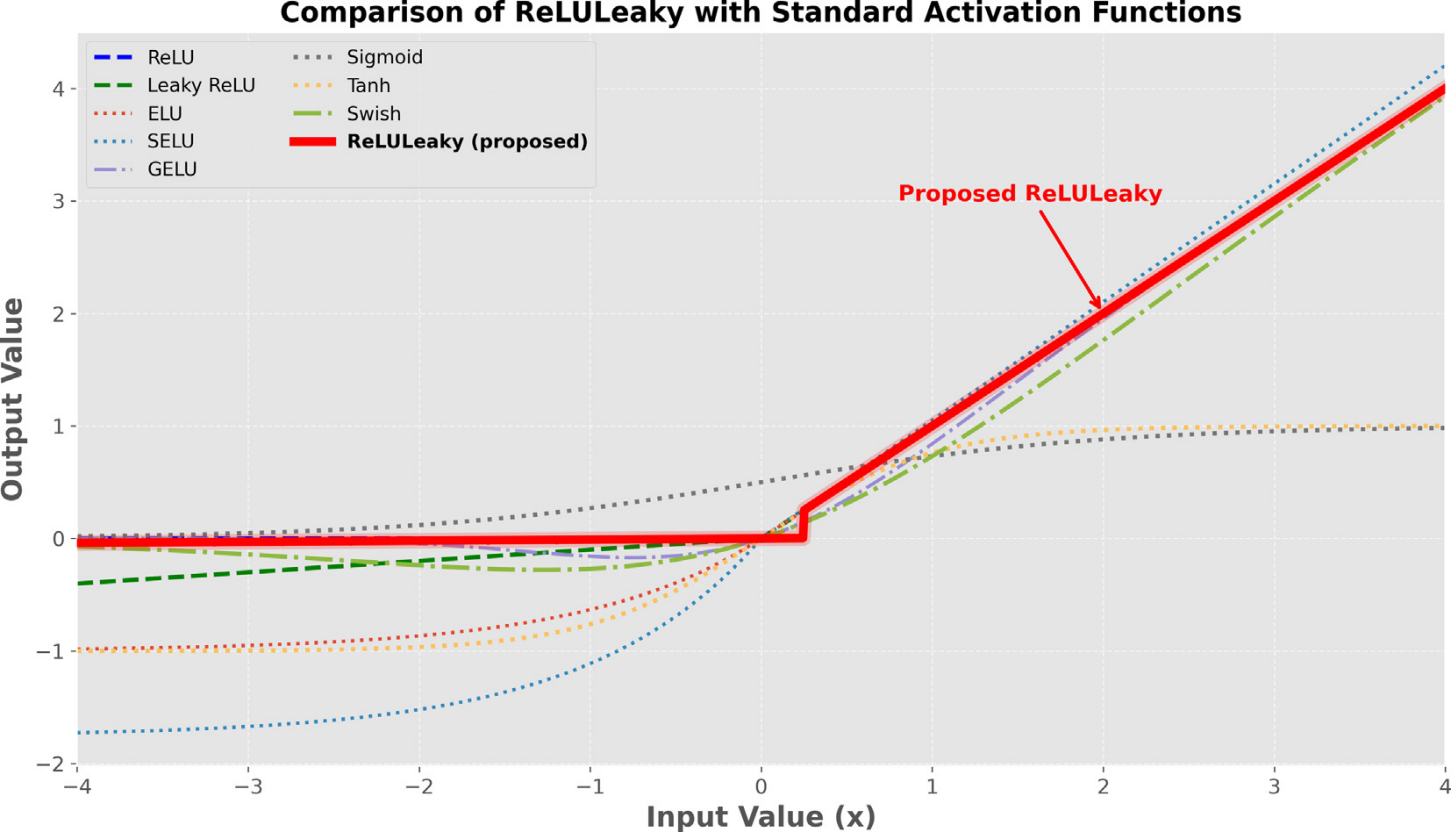
Comparison of the proposed ReLULeaky activation function with standard activation functions.

Fig. 8 illustrates a comparison between the proposed ReLULeaky activation function and several broadly used activation functions, including ReLU, Leaky ReLU, ELU, SELU, GELU, Sigmoid, Tanh, and Swish. The proposed ReLULeaky function combines the advantages of ReLU and Leaky ReLU by introducing a modest non-zero gradient for input values below the threshold while maintaining linear activation for larger positive inputs. Unlike the standard ReLU, which entirely suppresses negative activations and may suffer from the dying ReLU problem, the proposed function preserves gradient flow in the negative area, facilitating more stable optimization during training. As illustrated in the figure, ReLULeaky demonstrates a piecewise-linear behavior comparable to ReLU for positive inputs while preserving a controlled slope for lower activation values. This characteristic enables effective feature learning, improved gradient propagation, and enhanced robustness during deep neural network training.

### 3.7 Transfer learning

**Fig. 9:**
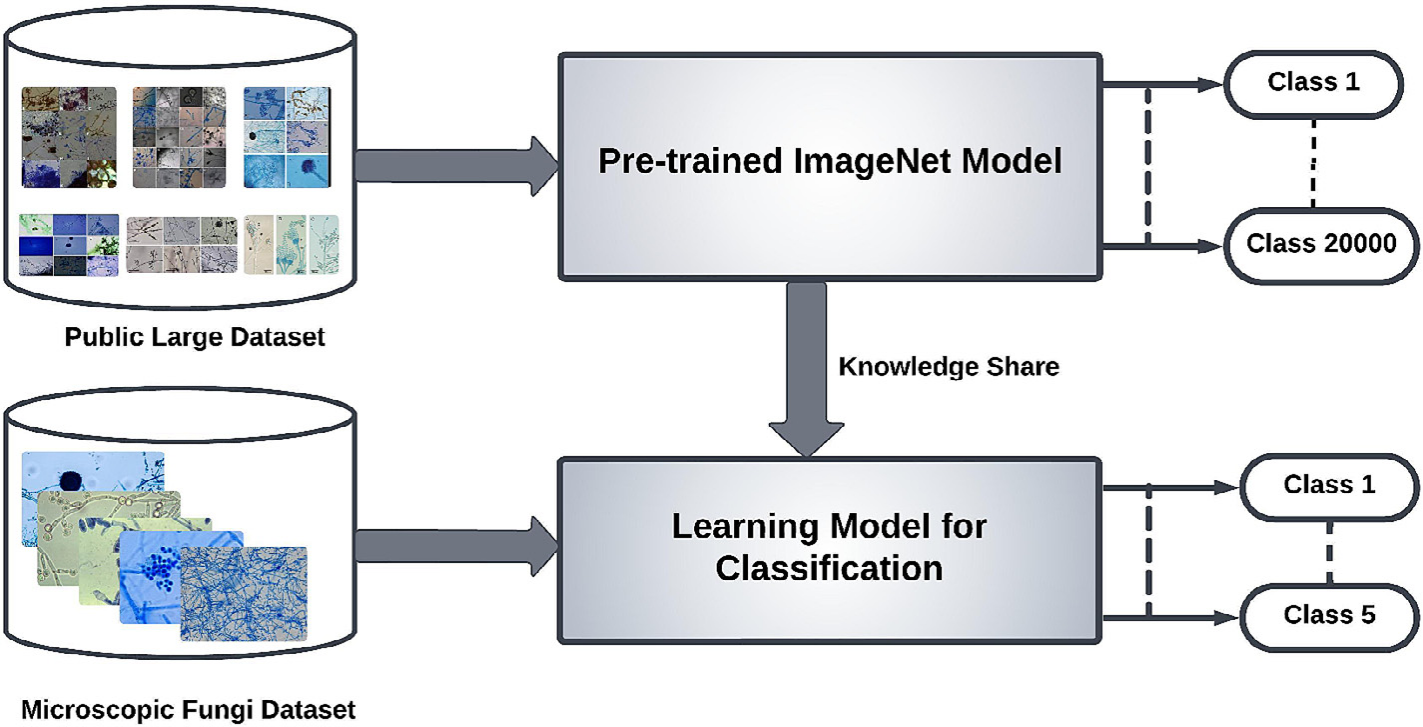
Transfer learning approach.

Transfer learning is a powerful approach in DL that leverages pretrained models on large datasets to solve specific, often smaller, tasks with limited data. In the context of microscopic fungi classification, transfer learning has proven to be particularly effective. Fig. 9 illustrates the transfer learning approach for microscopic fungi classification, leveraging a pretrained ImageNet model. The process involves two main stages: knowledge transfer from a pretrained model and task-specific fine-tuning. The top part of the diagram represents the initial stage where a large, publicly available dataset, such as ImageNet, is used to train a DL model. The dataset contains millions of images across thousands of classes (e.g., 20,000 categories).^34^ A model pretrained on this dataset learns to extract hierarchical features, such as edges, textures, and object structures, which are generalizable to many computer vision tasks. The weights and parameters learned during this training process are preserved for transfer to a new task. This stage is computationally expensive but results in a robust feature extractor. The bottom section represents the task-specific fine-tuning phase. Here, the pretrained model is adapted to classify microscopic fungi using a much smaller, domain-specific dataset containing images of fungi under a microscope.^35^ This dataset typically has fewer classes (e.g., five fungi species) and fewer images compared to the ImageNet dataset. The adaptation involves transferring the learned weights from the pretrained model to the new learning model.

### 3.8 GradCam method

GradCAM (Gradient-weighted Class Activation Mapping) is an effective technique used to visualize the areas of an input image that are considered most important to the decision-making process of a CNN.^36^ It obtains this by using the gradients of a specific class score concerning the feature maps of a convolutional layer, which are then weighted and combined to produce a heatmap over the input image.^37^

Mathematically, consider a CNN that processes an input image X and produces a score $y^{c}$ corresponding to a class c. Let the feature maps of a convolutional layer be denoted as $A^{k}$, where k indexes the feature maps, and $A^{k}$ has spatial dimensions h×w. The class score $y^{c}$ can be expressed as a function of $A^{k}$:

$$y^{c}=f\left(A^{1},A^{2},\ldots ,A^{K}\right).$$

The key idea behind GradCAM is to compute the gradient of the class score with respect to each feature map:

$$\frac{\partial y^{c}}{\partial A^{k}}.$$

These gradients reflect the corresponding contribution of each feature map item to the class score. GradCAM estimates the global average of these gradients over the spatial dimensions in order to generate an insightful visualization:

$$\alpha_{k}^{c}=\frac{1}{h\times w}\sum_{i=1}^{h}\sum_{j=1}^{w}\frac{\partial y^{c}}{\partial A_{\mathrm{ij}}^{k}}.$$

Here, $\alpha_{k}^{c}$ represents the importance weight for the feature map $A^{k}$ with respect to the class c. The final class activation map $L_{\text{GradCAM}}^{c}$ is obtained by a weighted combination of the feature maps:

$$L_{\text{GradCAM}}^{c}=\text{ReLULeaky}\left(\sum_{k}\alpha_{k}^{c}A^{k}\right).$$

The use of the proposed ReLULeaky function ensures that only features that have a positive impact on the class score are considered, thereby highlighting the areas of the input image that are most relevant to the class c. The resulting activation map $L_{\text{GradCAM}}^{c}$ is then upsampled to the size of the input image to create a heatmap that can be integrated on the image, providing an intuitive visualization of the model's focus regions.

**Fig. 10:**
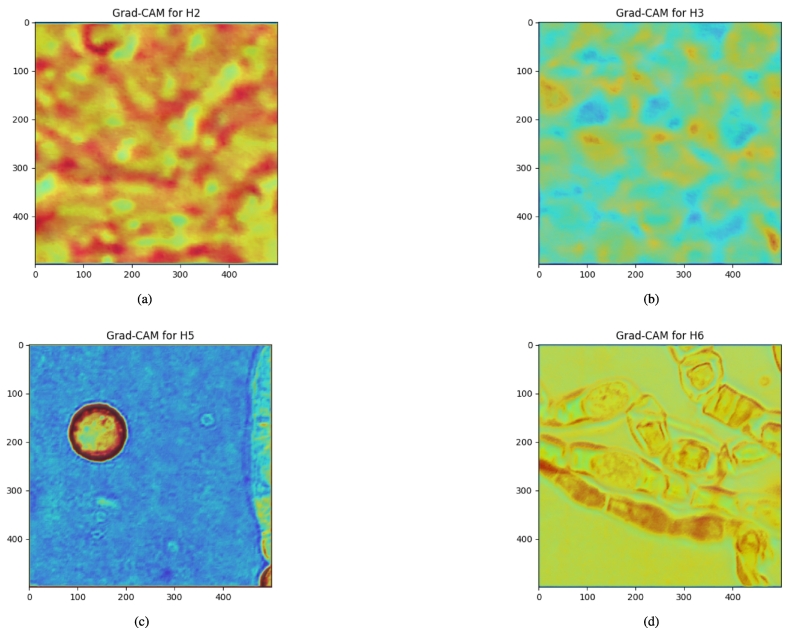
GradCAM-based interpretability visualizations of fungi classes.

Fig. 10 shows GradCAM insights into how the model focuses on different regions for four distinct fungi classes, labeled as H2, H3, H5, and H6. In H5, the scattered and intense activations suggest the model relies on multiple vague features, possibly reducing reliability. Weaker activations and diffuse patterns for H3 indicate it is difficult to isolate distinct features, which lowers confidence. In contrast, H2 shows strong, concentrated activations, meaning the model identifies distinct features to this class. H6 has the most structured and distinct patterns, indicating that the model can reliably identify fungi features, allowing for precise classification. Overall, these insights reveal class-wise differences in feature learnability and model certainty.

### 3.9 GradCAM++ method

GradCAM++ is a variation of the GradCAM method that improves on certain drawbacks, specially in cases where multiple instances of an object or finer details need to be visualized. While GradCAM is effective for generating class-specific visual explanations, it tends to produce fine heatmaps that may highlights on the most noticeable object within an image, often overlooking smaller or overlapping instances. GradCAM++ improves this process by refining how gradients are computed and aggregated, allowing for a more complex and detailed visualization.^38^ One of GradCAM++’s key innovations is its use of second-order gradients. While GradCAM computes importance weights based on the first-order gradients of the output class score with respect to the feature maps, GradCAM++ introduces a second-order term.

**Fig. 11:**
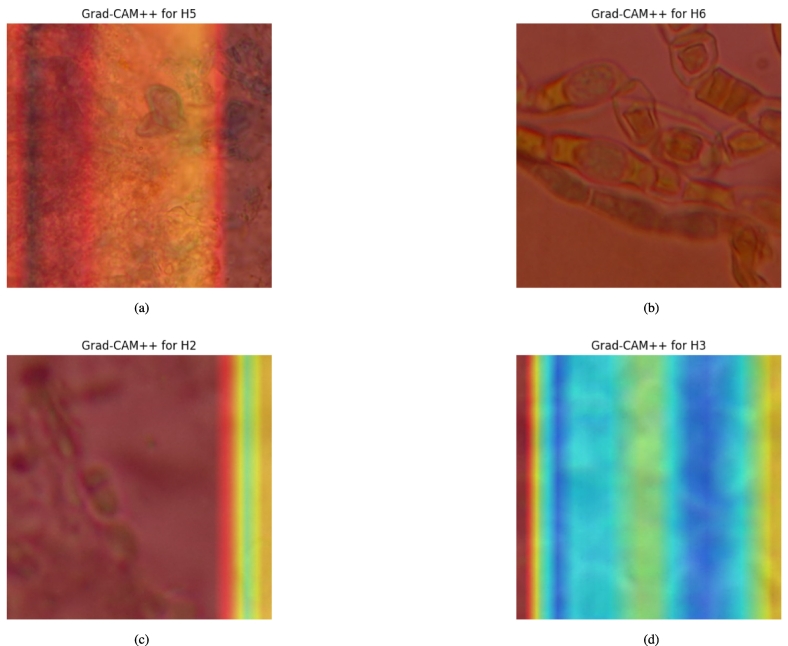
GradCAM++−based interpretability visualizations of fungi classes.

Fig. 11 depicts the GradCAM++ insights for four fungi classes. For class H5, the vertical red-orange activations indicate the model detects significant structural features along that axis. Class H6 shows precise, detailed activations over fungi structures, indicating high confidence and robust feature recognition. In class H2, the focus is confined to one side, meaning the model relies on certain, consistent features for classification. By contrast, class H3 shows diffuse, striped activations, reflecting uncertainty and less clear feature extraction. The visualizations highlight how the model's attention varies in clarity and focus across classes, influencing its classification confidence.

### 3.10 LIME method

LIME explains complex model predictions by estimating them locally with an easy, interpretable model. It divides the input image into superpixels using the SLIC method and then makes several modified versions by randomly masking some superpixels.^39^ The main model predicts each modified image, and their similarity to the original is used to weight them. A linear model is then fitted to these weighted samples to replicate the complex model's behavior near the input.^40^ The linear model's coefficients shows the importance of each superpixel in the prediction. This enables us to graphically understand which regions of the image most influenced the model's decision. Let x represent this image. Produce a set of modified samples by introducing occlusions or random noise into the image. We can accomplish this by masking certain areas of the image. A modified version of an image x can be expressed as $x^{\prime }=\text{Mask}\left(x\right)$. The modified images can be represented as:

$$X^{\prime }=\left\{x_{1}^{\prime },x_{2}^{\prime },\ldots ,x_{m}^{\prime }\right\}$$

where m is the number of altered image. For each altered image $x_{i}^{\prime }$, gain the model predictions $f\left(x_{i}^{\prime }\right)$. Assign weights to the modified samples based on their distance from the original instance. The weight for each alternation $x_{i}^{\prime }$ can be computed using a Gaussian kernel:

$$w_{i}=\exp\left(-\frac{d{\left(x,x_{i}^{\prime }\right)}^{2}}{\sigma^{2}}\right)$$

where $d\left(x,x_{i}^{\prime }\right)$ is a distance measure between the original image x and the perturbed image $x_{i}^{\prime }$. Use the perturbed images $X^{\prime }$ and their corresponding predictions $f\left(X^{\prime }\right)$ to train an interpretable model g:

$$g\left(z\right)=\beta_{0}+\sum_{j=1}^{p}\beta_{j}z_{j}$$

where z represents the features (e.g., superpixels) extracted from the altered images. The coefficients $\beta_{j}$ provide insights into which features (or superpixels) are important for the prediction.

LIME approximates the decision boundary of the complex model f around the instance x with the interpretable model g. The objective function for training g can be expressed as:

$$\min_{g}\sum_{i=1}^{m}w_{i}{\left(f\left(x_{i}^{\prime }\right)-g\left(z_{i}^{\prime }\right)\right)}^{2}+\Omega \left(g\right)$$

Where:

• $\Omega \left(g\right)$ is a regularization term.

**Fig. 12:**
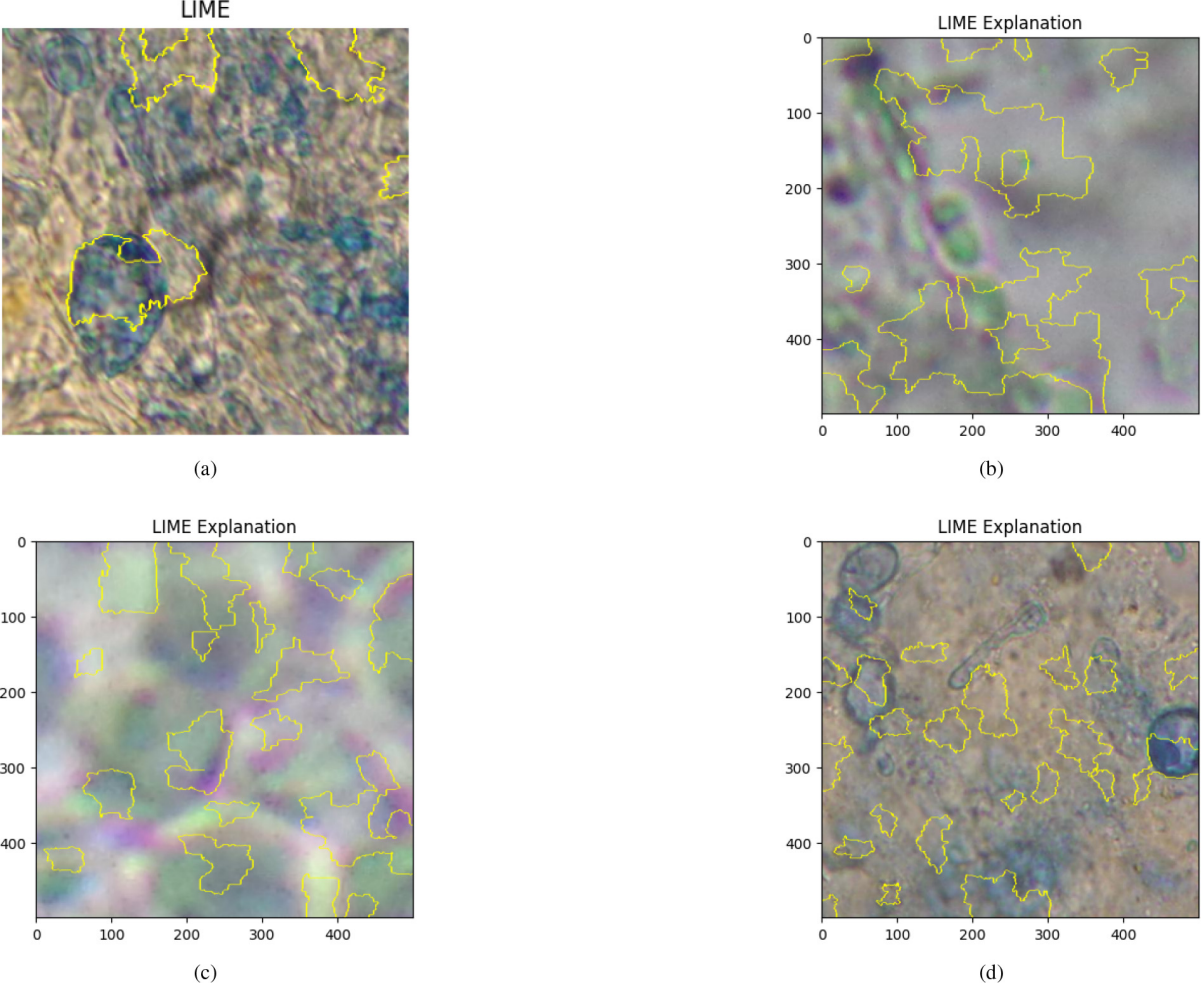
LIME-based interpretability visualizations of fungal classification results: (a) H2 (*Aspergillus niger*), (b) H3 (*Epidermophyton floccosum*), (c) H5 (*Trichophyton rubrum*), and (d) H6 (*Trichophyton mentagrophytes*).

Fig. 12 visualizations show how the model interprets different fungi classes. For H2 (Subfigure a), the model relies on broadly dispersed features, indicating less localized importance. H3 (b) shows focus on certain regions, indicating that distinct morphological features facilitate classification. H5 (c) has several important regions, meaning the model employs varied cues across the image. H6 (d) displays clear, well-structured segments highlighting fungi features, demonstrating strong recognition of distinct biological patterns.

### 3.11 FastAI library

The FastAI library is a comprehensive and high-level DL library built on top of PyTorch, designed to simplify and accelerate the development of DL models.^41^ It provides a suite of tools and modules that facilitate various stages of the machine learning pipeline, including data preprocessing, model training, and evaluation. With its intuitive DataBlock API, users can effortlessly handle complex data loading and augmentation tasks. The library supports transfer learning through access to a variety of pretrained models, which can be fine-tuned for specific tasks.^42^ The integrated Learner class encapsulates the model, data, and training loop, offering a seamless interface for training and experimentation. FastAI does not directly support splitting datasets into training, validation, and testing subsets, and is limited to a train–validation split. As a result, experiments conducted with FastAI followed this two-way splitting approach. To enable a more comprehensive evaluation, a three-way train–validation–test split was implemented using the TensorFlow library. FastAI also includes a range of callbacks and metrics for detailed monitoring and performance evaluation. FastAI is an invaluable resource for newcomers and experienced DL practitioners, enabling rapid development and deployment of cutting-edge models.

## 4 Results and discussion

The model's performance is thoroughly assessed using a variety of metrics and visualizations in this results section. By depicting the distribution of accurate and inaccurate predictions across many classes, the confusion matrix offers information on classification accuracy. Plotting the true-positive rate against the false-positive rate yields the receiver operating characteristic (ROC) curve, which shows how well the model can differentiate between classes. The classification report also provides a comprehensive evaluation of the model's prediction skills by summarizing important performance parameters, including accuracy, recall, F1-score, and support. Besides, a comparative study of past research is explained in this section.

### 4.1 Experimental setup

The proposed fine-tuned ResNet34 model was trained to classify five fungi classes using the FastAI framework, along with tools like Python, TensorFlow, Kaggle's GPU P100 support, and a computer (2.30 GHz Intel Core i3, 8746 U CPU, 8GB RAM). Training was performed for 32 epochs with a learning rate of 0.002 and a batch size of 32, ensuring a balance between speed and accuracy. A proposed ReLULeaky activation with a learnable threshold was incorporated to improve feature extraction. The AdamW optimizer was applied to manage weight decay and improve generalization. Furthermore, FastAI's one-cycle learning rate policy was used to speed up convergence. This configuration led to efficient learning and improved model performance for fungi classification.

### 4.2 Performance metrics

**Fig. 13:**
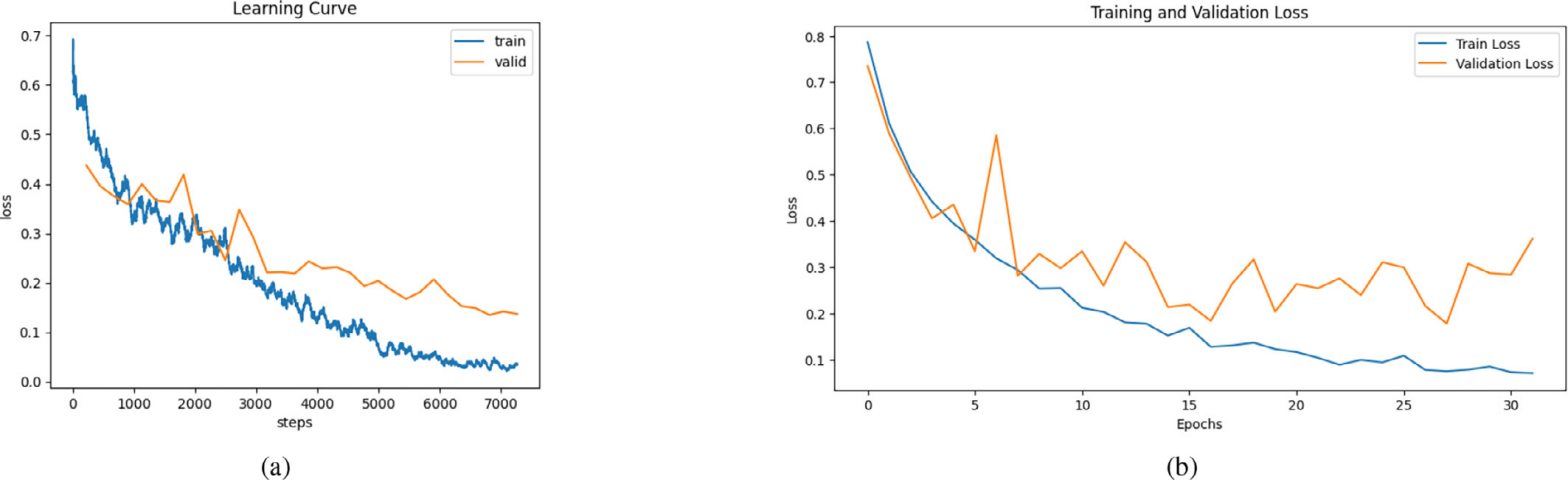
Loss curve (subfigure: (a) using the FastAI library's implementation (b) using the TensorFlow library's implementation).

Performance metrics are crucial to evaluate how accurately and consistently a model identifies different fungi classes. Fig. 13 shows how training and validation loss change over time during the fungi classification model training using the FastAI and Tensorflow library's implementation. Initially, both losses are high, indicating poor performance, as the model is either randomly initialized or pretrained but not yet fine-tuned. As training progresses, the training loss quickly decreases, showing that the model is effectively learning key features of the fungi images. The validation loss helps monitor how well the model generalizes to unseen data. The early sharp drop in training loss highlights the model's ability to learn foundational image patterns.

The model effectively learns to identify fungi images based on essential visual patterns when both curves converge toward low loss values without substantially diverging, indicating excellent training without overfitting. It also initially reduces the validation loss, which is calculated on a different set of unseen data. It demonstrates that the learnt features of the model in the early training phase transfer effectively to the validation dataset. As the model gets better at making predictions on both visible and unseen data, the simultaneous decline in training and validation losses shows that learning is occurring. The validation loss's rate of collapse, however, is slower than the training loss's, which is typical given that the validation data contains more noise and variability for the model.

**Fig. 14:**
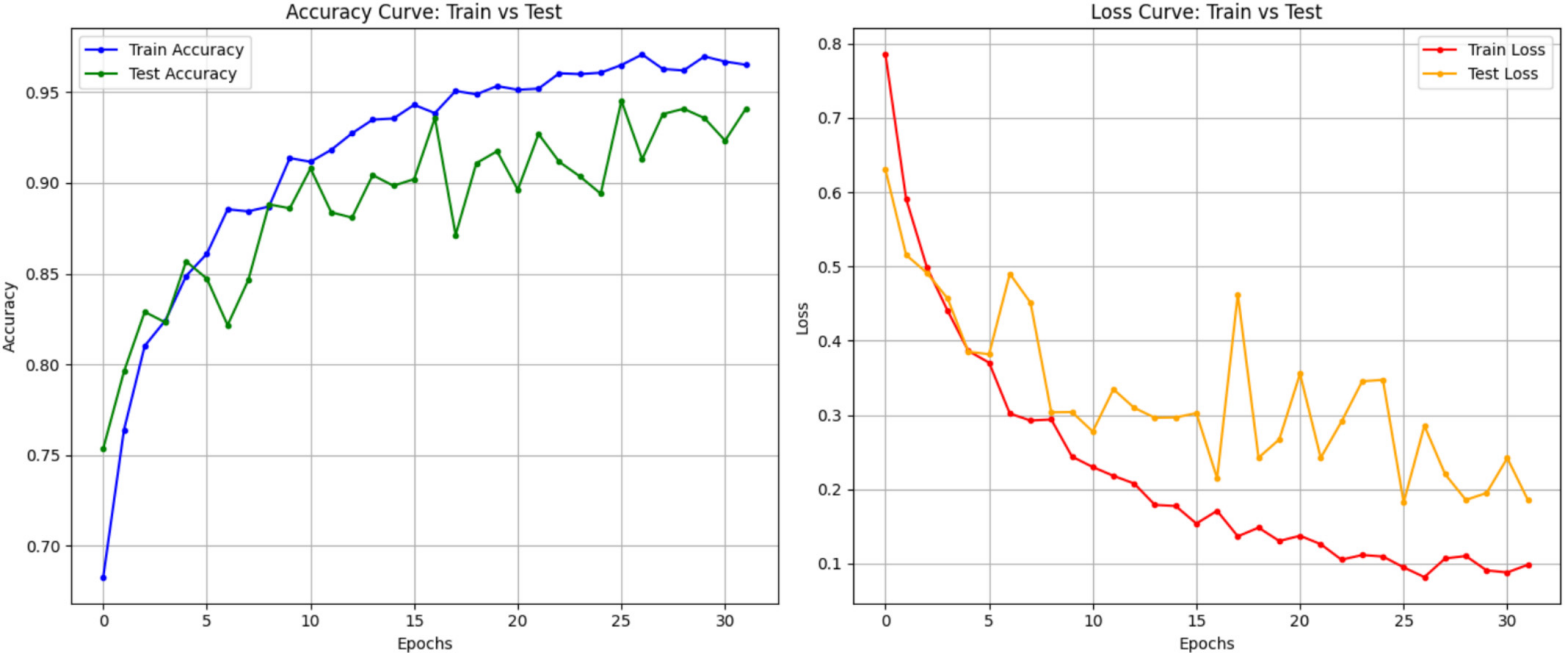
Train vs Test curve (using the TensorFlow library's implementation).

Fig. 14 shows the training and test performance of the proposed fungi classification model implemented using the TensorFlow library with a three-way data split. The accuracy curve demonstrates a consistent improvement in training accuracy, indicating effective learning of discriminative fungal features. The test accuracy follows a similar trend and stabilizes at a high level, reflecting good generalization on unseen samples. The loss curve shows a steady decrease in training loss, confirming stable convergence during optimization. Although minor fluctuations are observed in the test loss, the overall downward trend suggests robust model learning. These results validate the effectiveness of the three-way data splitting strategy for reliable fungi classification.

**Fig. 15:**
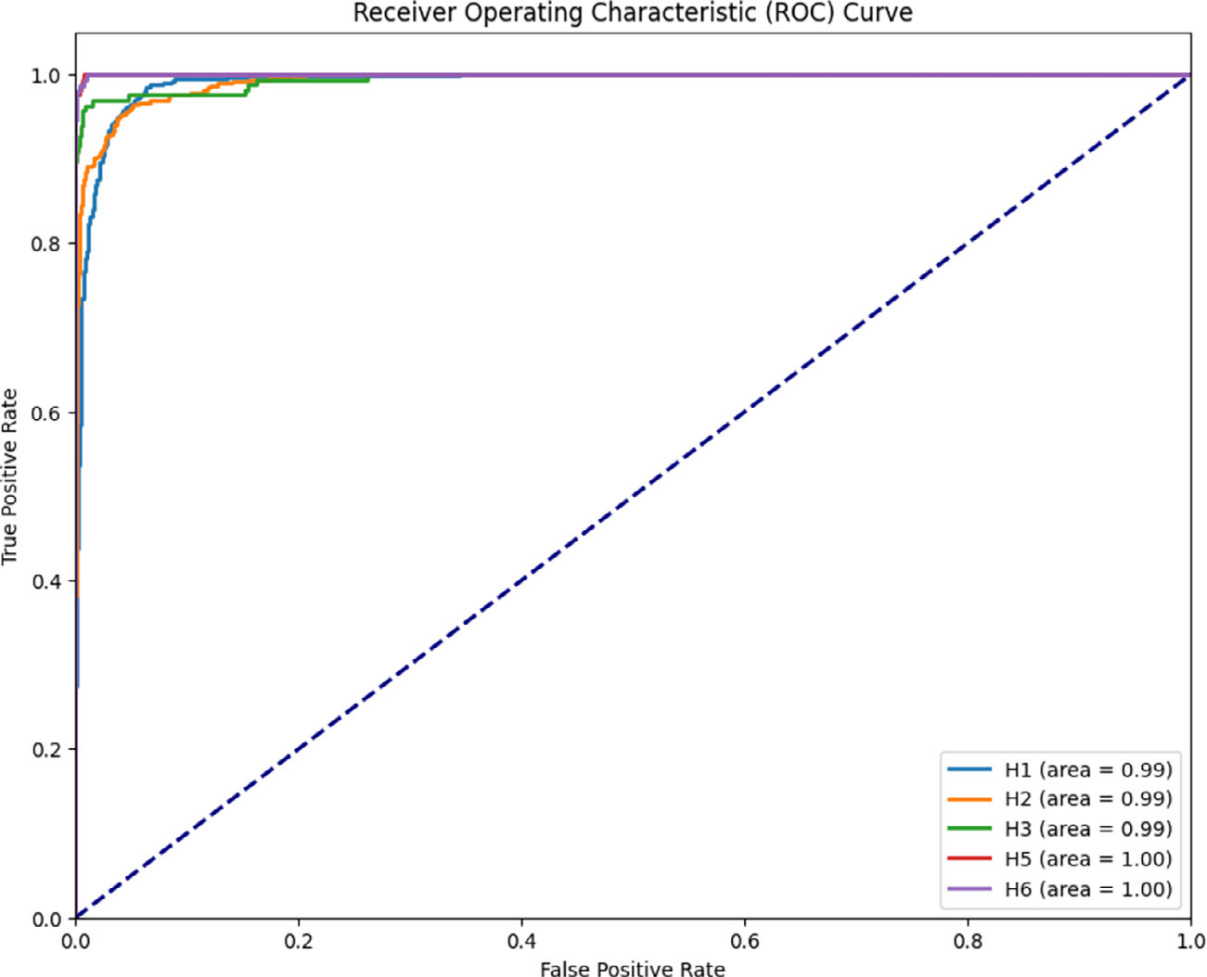
ROC curve.

Fig. 15, the ROC curve, depicts a model's classification performance for fungal classification tasks with five distinct classes, shown as H1, H2, H3, H5, and H6. A stochastic classifier would produce decisions with little predictive power, as seen by the diagonal dashed line in the plot. The ROC curve is above this baseline, which indicates the model is outperforming random guessing. The classifier's ability to discriminate between the five fungi classes is demonstrated by the curves for each class being substantially above the diagonal line.

A single scalar measurement known as the area under the curve (AUC) measures the classifier's overall effectiveness for every class. Every ROC curve for the fungi classes almost reaches the top-left corner, indicating flawless classification performance. A confusion matrix is a primary method for evaluating classification models by comparing actual class labels with predicted labels. In multi-class classification, the confusion matrix extends to a C×C table, where each entry $M_{\mathrm{ij}}$ represents the number of times an instance of class i was predicted as class j.

Several key performance metrics can be derived from the confusion matrix. Accuracy measures the overall correctness of the model:

$$\text{Accuracy}=\frac{\mathrm{TP}+\mathrm{TN}}{\mathrm{TP}+\mathrm{TN}+\mathrm{FP}+\mathrm{FN}}$$

Where TP and TN denote true positive and true negative. FP and FN mean false positive and false negative instances. Precision determines how many of the predicted positive cases are actually positive.

$$\text{Precision}=\frac{\mathrm{TP}}{\mathrm{TP}+\mathrm{FP}}.$$

Recall or sensitivity (TP rate), evaluates the model's ability to correctly identify positive instances:

$$\text{Recall}=\frac{\mathrm{TP}}{\mathrm{TP}+\mathrm{FN}}.$$

A balance between precision and recall is captured using the F1-score, which is the harmonic mean of both.

$$F1=2\times \frac{\text{Precision}\times \text{Recall}}{\text{Precision}+\text{Recall}}.$$

Additionally, specificity (TN rate) measures the ratio of correctly identified negative cases.

$$\text{Specificity}=\frac{\mathrm{TN}}{\mathrm{TN}+\mathrm{FP}}.$$

**Fig. 16:**
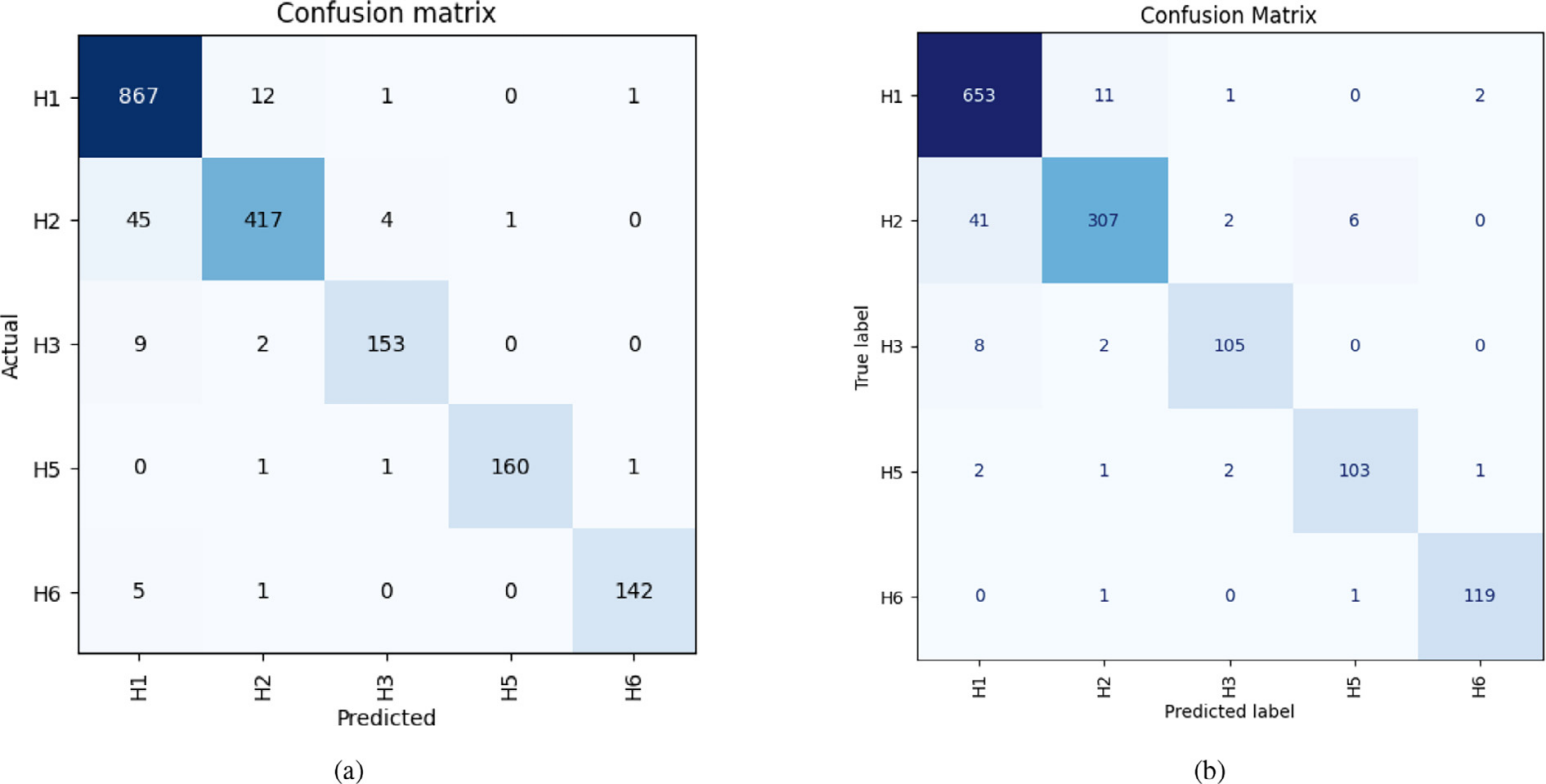
Confusion matrix (subfigure: (a) using the FastAI library's implementation (b) using the TensorFlow library's implementation).

Fig. 16 shows the confusion matrix of a fungi classification model across five classes. Each row corresponds to the actual class, whereas each column corresponds to the predicted class. Subfigure 16a demonstrates strong classification performance of the FastAI-based fungi model under the train–validation scheme. Class H1 shows the strongest performance, with 867 images correctly classified, and only a small number misclassified mainly as H2, suggesting slight visual similarity between these 2 classes. Similarly, H2 achieves a high number of correct predictions (417), with some confusion primarily toward H1, which may be due to overlapping microscopic morphological features. Classes H3, H5, and H6 demonstrate excellent discrimination, with very few misclassifications and high diagonal values (153, 160, and 142, respectively). The minimal off-diagonal entries for these classes indicate that the model effectively captures their distinctive visual patterns. Subfigure 16b shows the comparatively lower recognition accuracy on the train,test, and validation splittings scheme. Class H1 exhibits the highest recognition accuracy, with 653 correctly classified samples and minimal misclassification, primarily into H2, suggesting slight visual similarity between these 2 classes. Class H2 shows comparatively higher confusion, particularly with H1, where 41 samples are misclassified. Classes H3, H5, and H6 display robust separability, each achieving high TP rates with negligible cross-class errors, reflecting well-learned discriminative representation.

Table 1 depicts the strong and balanced performance of the proposed fungi classification model across all classes. Hence, accuracy reaches 95%, demonstrating reliable generalization. Class-wise results show high precision and recall for most classes, with H5 and H6 achieving near-perfect scores, indicating excellent separability. H1 exhibits very high recall (0.98), confirming effective detection of this class, whereas H2 shows slightly lower recall (0.89), suggesting occasional confusion with other fungi types. The macro-averaged F1-score (0.96) reflects consistent performance across classes, and the weighted average (0.95) confirms robustness despite class imbalance. Table 2 shows that the highest precision, recall, and F1-score are 0.98 for class H6. The overall test accuracy achieves 0.94, which is the lower than FastAI library's implementation schemes.

**Table 1:** Classification report for fungi classes (using FastAI library's implementation).

Class	Precision	Recall	F1-score	Support
H1	0.94	0.98	0.96	881
H2	0.96	0.89	0.93	467
H3	0.96	0.93	0.95	164
H5	0.99	0.98	0.99	163
H6	0.99	0.96	0.97	148
Accuracy	**0.95**			1823
Macro Avg	0.97	0.95	0.96	1823
Weighted Avg	0.95	0.95	0.95	1823

**Table 2:** Classification report for fungi classes (using Tensorflow library's implementation).

Class	Precision	Recall	F1-score	Support
H1	0.93	0.98	0.95	667
H2	0.95	0.86	0.91	356
H3	0.95	0.91	0.93	115
H5	0.94	0.94	0.94	109
H6	0.98	0.98	0.98	121
Accuracy	**0.94**			1368
Macro Avg	0.95	0.94	0.94	1368
Weighted Avg	0.94	0.94	0.94	1368

**Fig. 17:**
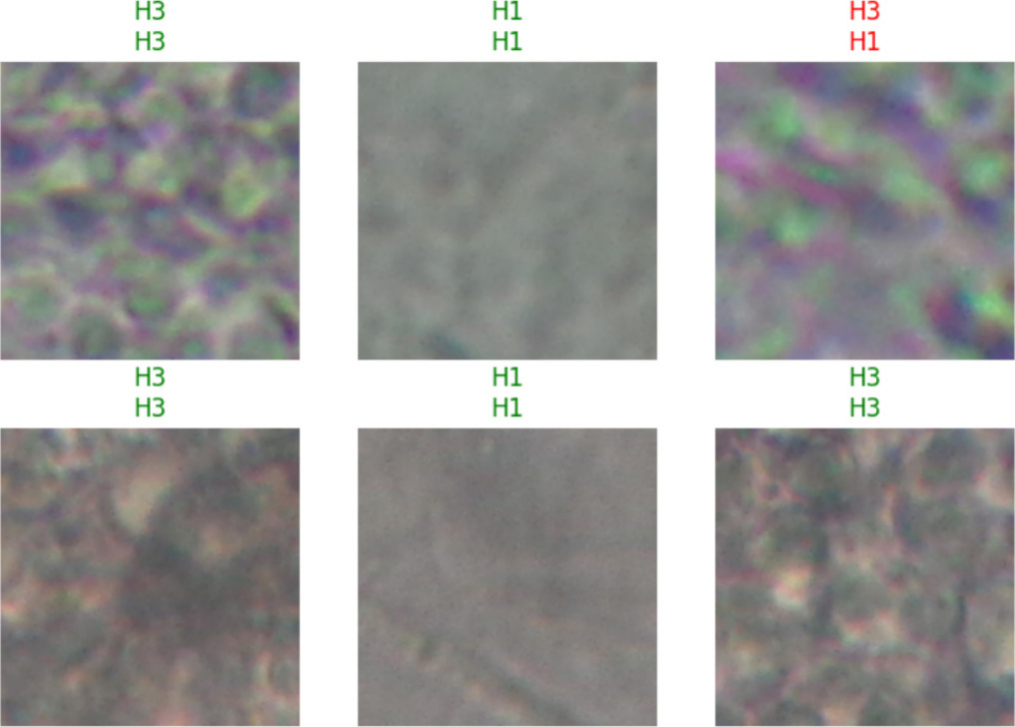
Prediction of randomly selected images.

Fig. 17 shows a collection of randomly chosen fungi images with their predicted and actual labels. Each image is annotated with the predicted label on top and the actual label below, both in green for all classes except class H3, which is a false prediction. The model has correctly classified all the fungi images, as the predicted and actual labels match for each image. This shows that the model is performing well not only on the confusion matrix level but also in individual image-level predictions, showing reliable classification across different fungi classes.

### 4.3 K-fold cross-validation

Table 3 summarizes the performance of the proposed model evaluated using 5-fold cross-validation. The dataset was divided into five equal subsets, where each fold was used once as a validation set, whereas the remaining four folds were used for training. The model achieves consistently high accuracy and F1-score across all folds, indicating stable and reliable performance. The mean accuracy and F1-score of approximately 95% demonstrate strong classification capability, whereas the low standard deviation (0.0055) reflects minimal performance variation between folds.

**Table 3:** Performance of the ReLULeaky-ResNet34 model using 5-fold cross-validation.

Fold	Accuracy	F1-score
Fold 1	0.9435	0.9433
Fold 2	0.9462	0.9458
Fold 3	0.9512	0.9511
Fold 4	0.9594	0.9592
Fold 5	0.9523	0.9520
Mean	**0.9505**	**0.9503**
Std. deviation	**0.0055**	**0.0055**

**Fig. 18:**
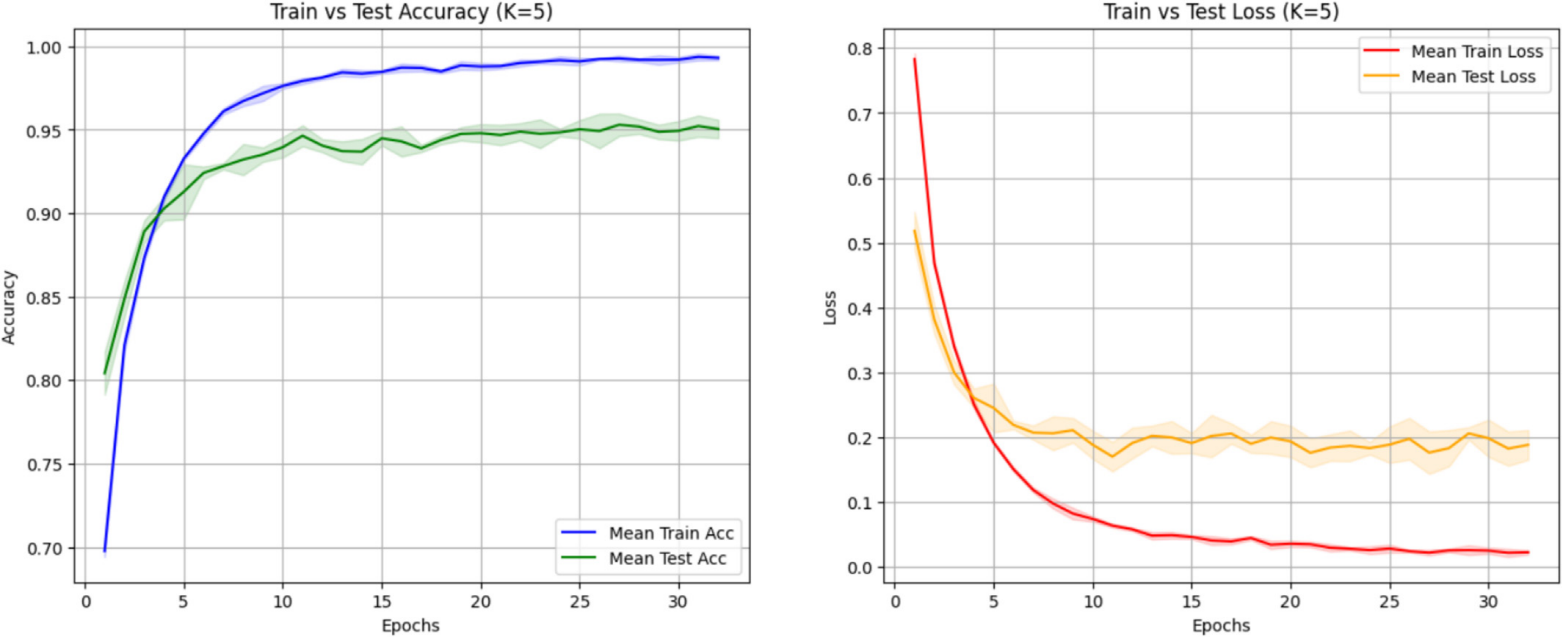
5-fold cross-validation curve.

5-fold cross-validation curves (Fig. 18) demonstrate a highly robust and accurate model, achieving a mean test accuracy of approximately 95% against a training accuracy of nearly 99%. The narrow shaded regions (standard deviation) on both graphs indicate consistent performance across all five folds, proving the model is stable and not biased toward specific data splits. Whereas the gap between the training and test lines suggests mild overfitting, the Loss curve confirms the model behaves well. The test loss flattens out around 0.2 by epoch 15 rather than spiking upward, indicating the model has successfully converged and generalizes effectively to new fungi classes.

### 4.4 Performance analysis of model

The comparison of several DL models is shown in this Table 4 using three important performance metrics as accuracy (%), precision (%), and F1-score (%). There are some semi-supervised models and supervised DL models included into the comparison table.

**Table 4:** Comparative performance analysis of different deep learning models.

Method	Accuracy (%)	F1-score (%)	Precision (%)
EfficientNetB3	88.43	89.00	89.00
GoogleNet	64.23	62.00	66.00
XceptionNet	88.37	89.00	91.00
MobileNet	36.64	47.00	59.00
ResNet50	82.89	84.00	87.00
DenseNet121	74.55	77.00	78.00
VGG16	68.26	68.00	65.00
FixMatch	58.02	58.00	57.00
Mean teacher	48.00	49.00	48.00
MixMatch	61.05	59.00	60.00
ReLULeaky-ResNet34 (Tensorflow)	94.08	94.00	0.95
ReLULeaky-ResNet34 (FastAI)	**95.39**	**96.00**	**97.00**

Fine-tuned ReLULeaky-ResNet34 model tops all other models with the highest accuracy, precision, and F1-score of 95.39%, 97%, and 96%, respectively (using the FastAI implementation). The accuracy, F1-score, and precision of our proposed model achieve 94.08%,94%, and 95%, respectively, using the implementation of the TensorFlow library. Mean Teacher and MobileNet, on the other hand, perform the worst, with accuracy rates of 48.00% and 36.64%, respectively. Although the ReLULeaky-ResNet34 model is currently superior to them, the outstanding accuracy of XceptionNet (88.37%) and EfficientNetB3 (88.43%) indicates their incredible classification knowledge. This comparison shows that ReLULeaky-ResNet34 is the best model for the classification task at together, which makes it the most suitable choice for deployment.

Table 5 shows a comparison of different DL models according to their classification accuracy. The methods such as BiT, MobileNetV3, ResNet variants (ResNet50, ResNet101), DenseNet121, ViT with ResNet50, Mfungx, and ORDAF are included in the existing study.

**Table 5:** Comparison between the proposed method and existing studies.

Method	Accuracy (%)
BiT method (Big transfer)^30^	87.32
MobileNetV3^43^	92.89
ResNet50^18^	85.00
DenseNet121^44^	91.01
ResNet101^45^	93.00
Mfungx^27^	92.49
VIT + ResNet50^29^	90.13
ORDAF^46^	77.44
ReLULeaky-ResNet34	**95.39**

Fine-tuned ReLULeaky-ResNet34^45^ model outperforms the other models in classification, as shown by its maximum accuracy of 95.39%. The good performance is also shown by Mfungx (92.49%), MobileNetV3 (92.89%), and ResNet101 (93.00%). The accuracy of ORDAF (77.44%) and the BiT approach (87.32%) is somewhat lower. ReLULeaky-ResNet34 is the most feasible model for the specified classification task, as shown by this comparison.

Table 6 shows the comparison among different activation functions on the performance of the ResNet34 model. Among the tested functions, ReLULeaky obtains the highest performance, with an accuracy of 95.39%, F1-score of 96.00%, and precision of 97.00%, making it the most effective standalone activation function. Leaky ReLU, Elu, and Selu also demonstrate strong results, each achieving over 90% in most metrics, indicating their suitability for DL-based fungi classification. Applying different solo activation functions, we can see the visual impact on the activation function of the model. The above table shows the ups and downs of the model's performance based on the activation function.

**Table 6:** Comparison among different activation functions of the ResNet34 model.

Activation function	Accuracy (%)	F1-score (%)	Precision (%)
Sigmoid	68.73	64.00	67.00
ReLU	93.92	94.00	94.00
Selu	88.69	91.00	93.00
Elu	90.56	92.00	94.00
Leaky ReLU	93.22	93.00	94.00
Gelu	90.83	92.00	93.00
Swish	88.59	90.00	92.00
ReLULeaky	**95.39**	**96.00**	**97.00**

Table 7 shows the performance evaluation of different proposed activation functions integrated into a fine-tuned ResNet34 model. The authors develop several hybrid proposed activation functions to evaluate the robustness and efficiency of the model. Among them, the RelLeaky with Learnable Threshold function significantly outperforms all others, achieving the highest accuracy (95.39%), F1-score (96.00%), and precision (97.00%). To verify its efficacy, the authors compare this proposed ReLULeaky activation function with other hybrid activation functions such as ReLU with Leaky ReLU, ReLU with ELU, and Leaky ReLU variants like LRDT and LRS. Although ReLU with Leaky ReLU, and ReLU with Elu also show strong performance (accuracy greater than 90%), they fall short of the proposed function. The poor performance of Leaky Softmax (accuracy 49.42%) highlights its unsuitability for this task. Overall, the results show that the proposed ReLULeaky with Learnable Threshold activation function substantially enhances the classification capabilities of the fine-tuned ResNet34 model for fungi classification.

**Table 7:** Performance comparison of different custom activation functions for fine-tuned ResNet34 model.

Custom activation function	Accuracy (%)	F1-score (%)	Precision (%)
Sigmoid + Tanh	85.90	87.00	89.00
Leaky ReLU with dynamic threshold (LRDT)	90.80	92.00	93.00
Leaky ReLU with Swish (LRS)	86.34	87.00	90.00
Leaky Softmax	49.42	17.00	19.00
ReLU with Leaky ReLU	93.95	94.00	95.00
ReLU with Elu	90.56	92.00	93.00
ReLU with Selu	86.45	88.00	91.00
ReLULeaky with learnable threshold	**95.39**	**96.00**	**97.00**

Table 8 compares the impact of different thresholding strategies on the performance of the ReLULeaky activation function. The layer-wise learnable threshold attains the highest accuracy (95.39%), F1-score (96%), and precision (97%), indicating superior adaptability. Fixed thresholds perform the worst, highlighting the importance of dynamic thresholding. Other methods like EMA and adaptive scaling also yield competitive results but fall slightly short of the layer-wise approach. The experimental results analysis demonstrates that the proposed fine-tuned ReLULeaky-ResNet34 model, enhanced with a proposed ReLULeaky activation function and optimized training strategies, attains high accuracy and robustness in classifying fungi images. The model consistently performs well across all classes, as evidenced by near-perfect AUC scores, high precision, recall, and F1-scores. Overall, the approach offers a reliable and interpretable solution for automated fungi classification, paving the way for its practical application in biological and medical domains.

**Table 8:** Impact of threshold value on the proposed ReLULeaky activation function.

Threshold	Accuracy (%)	F1-score (%)	Precision (%)
Learnable threshold (layer-wise value)	**95.39**	**96.00**	**97.00**
Global learnable threshold	94.35	95.00	97.00
Fixed threshold	76.96	78.00	85.00
Mean of input	91.27	92.00	93.00
Exponential moving average (EMA)	94.62	95.00	96.00
Adaptive threshold with learnable scaling factor	92.59	94.00	95.00

Table 9 presents a comparative evaluation of the proposed ReLULeaky activation function against several standard activation functions across multiple medical image classification datasets.^53^ The results demonstrate that the proposed ReLULeaky function consistently obtains superior or very competitive F1-score and precision values across most classes and datasets. In particular, ReLULeaky attains near-perfect performance in various classification tasks, indicating its effectiveness in preserving discriminative features and facilitating robust gradient propagation. The learnable threshold method enables adaptive feature activation, contributing to enhanced classification performance compared with conventional fixed-threshold activation functions. The findings highlight the generalization capability and effectiveness of the proposed ReLULeaky activation function across diverse medical imaging applications. Although the proposed ReLULeaky-ResNet34 model achieved promising performance across fungal species and multiple medical image datasets, several drawbacks remain. First, the ResNet34 model may not capture more complex features for different datasets. Second, whereas train–validation–test splitting and 5-fold cross-validation were performed to reduce potential bias and data leakage, more validation on external independent datasets is needed. Third, the proposed ReLULeaky function was compared with various standard and custom activation functions. However, evaluation against a broader range of current adaptive activation functions could provide a more thorough assessment. Finally, the explainability studies were primarily qualitative and would benefit from expert-driven validation by mycologists or pathology specialists.

**Table 9:** Performance comparison of different activation functions on different datasets.

Datasets	Classes	Proposed ReLULeaky		Sigmoid		Tanh		ReLU		SeLU		ELU		GELU		Leaky ReLU	
		F1	Precision	F1	Precision	F1	Precision	F1	Precision	F1	Precision	F1	Precision	F1	Precision	F1	Precision
Alzheimer's disease dataset^47^	Mild demented	0.98	0.99	0.60	0.54	0.50	0.68	0.97	0.98	0.96	0.94	0.89	0.87	0.98	0.99	1.00	0.99
	Moderate demented	1.00	1.00	0.62	0.54	0.58	0.73	1.00	1.00	0.98	0.99	0.96	0.94	0.98	0.97	1.00	0.98
	Non-demented	1.00	1.00	0.97	0.25	0.44	0.60	0.98	0.98	0.96	0.99	0.88	0.84	0.99	0.97	0.98	0.98
	Very mild demented	0.99	0.99	0.40	0.99	0.55	0.91	0.97	0.97	0.98	0.99	0.88	0.91	0.99	1.00	0.99	0.99

Brain tumor dataset^48^	Glioma	1.00	1.00	0.97	0.96	0.91	0.91	0.99	0.98	0.96	0.94	0.89	0.94	0.99	1.00	1.00	1.00
	Meningioma	0.99	1.00	0.92	0.93	0.93	0.89	1.00	0.98	0.93	0.94	0.96	0.99	0.99	0.96	0.99	1.00
	Pituitary	1.00	0.99	0.96	0.96	0.94	0.96	0.99	1.00	0.93	0.96	0.96	0.90	0.88	0.98	0.99	0.97

Breast cancer dataset^49^	Benign	1.00	1.00	0.99	0.96	0.88	0.95	1.00	0.99	1.00	1.00	0.80	0.95	0.94	0.97	0.99	0.97
	Malignant	1.00	1.00	0.95	0.96	0.96	0.98	1.00	1.00	1.00	1.00	0.90	0.93	0.96	0.95	0.98	1.00

Cervical cancer dataset (SipakMed)^50^	Dyskeratotic	0.98	1.00	0.92	0.94	0.92	0.94	0.99	0.97	0.99	0.99	0.88	0.94	0.96	0.96	0.99	1.00
	Koilocytotic	0.99	0.99	0.91	0.88	0.92	0.89	0.98	1.00	0.96	0.93	0.91	0.90	0.98	0.95	0.98	0.99
	Metaplastic	0.99	1.00	0.94	0.94	0.96	0.93	0.99	0.98	0.99	0.99	0.91	0.93	0.90	0.98	0.98	1.00
	Parabasal	1.00	1.00	0.99	0.99	0.96	0.99	0.98	1.00	1.00	1.00	0.86	0.90	0.97	0.98	0.99	1.00
	Superficial intermediate	1.00	0.99	0.94	0.95	0.99	0.95	0.99	1.00	0.99	0.98	0.89	0.94	0.97	0.99	0.99	0.99

CT kidney dataset^51^	Normal	0.99	0.98	0.99	0.98	0.96	0.95	1.00	1.00	1.00	0.99	0.99	0.98	0.99	0.99	1.00	1.00
	Tumor	0.99	1.00	0.99	0.99	0.92	0.97	1.00	0.99	0.99	0.99	0.90	0.95	0.97	0.97	0.99	1.00

Lung and colon cancer dataset^52^	Lung benign tissue	1.00	1.00	0.93	0.92	0.90	0.93	0.98	0.98	0.99	0.96	0.79	0.89	0.96	0.94	0.98	0.98
	Lung adenocarcinoma	0.99	1.00	0.92	0.93	0.98	0.96	0.98	1.00	0.99	1.00	0.89	0.92	0.97	0.94	1.00	0.98
	Lung squamous cell carcinoma	0.99	1.00	0.88	0.89	0.78	0.86	1.00	0.98	0.96	1.00	0.90	0.92	0.97	0.96	0.98	1.00
	Colon adenocarcinoma	1.00	0.99	0.99	0.99	0.91	0.96	0.99	0.99	0.99	0.93	0.92	0.94	0.93	0.96	0.96	0.98
	Colon benign tissue	1.00	0.99	0.91	0.90	0.89	0.93	1.00	0.99	0.98	1.00	0.90	0.92	0.98	0.95	0.99	1.00

## 5 Conclusion

Given the prior importance of time and accurate diagnosis of fungal infection, we have developed and thoroughly evaluated a DL pipeline for automated fungi classification in this study. We obtained state-of-the-art performance across five fungal classes by fine-tuning a ResNet-34 backbone with a proposed ReLULeaky activation function comprising a learnable threshold with an overall 95.39% of accuracy and an average 99.4% of AUC scores. Comprehensive data preprocessing, data augmentation, and class balancing ensured robust feature learning, whereas AdamW optimization facilitated rapid convergence and consistent generalization. Our model's focus on biologically significant structures was validated by interpretability tests using GradCAM, GradCAM++, and LIME, which increased confidence in its predictions. According to experimental data, our method performs superiorly on fungi classification tasks compared to traditional CNN models like VGG16, ResNet50, and InceptionV3. All together, these results show that our method is a promising tool for speeding up mycological diagnostics and furthering research in medical and agricultural mycology because it not only excels in predicting accuracy but also provides precise, explainable insights.

## Declaration of competing interest

The authors declare that they have no conflict of interest.
